# Bariatric Surgery can Lead to Net Cost Savings to Health Care Systems: Results from a Comprehensive European Decision Analytic Model

**DOI:** 10.1007/s11695-014-1567-5

**Published:** 2015-02-02

**Authors:** Oleg Borisenko, Daniel Adam, Peter Funch-Jensen, Ahmed R. Ahmed, Rongrong Zhang, Zeynep Colpan, Jan Hedenbro

**Affiliations:** 1 Synergus AB, Svardvagen 19, 182 33 Danderyd, Sweden; 2 Aarhus University, and Aleris Hamlet Hospital Aarhus, Aarhus, Denmark; 3 Imperial College London, London, UK; 4 Aleris Obesity & Lund University, Lund, Sweden

**Keywords:** Bariatric surgery, Cost-utility analysis, Cost-effectiveness analysis, Health economics, Cost, Sweden

## Abstract

**Background:**

The objective of the present study was to evaluate the cost-utility of bariatric surgery in a lifetime horizon from a Swedish health care payer perspective.

**Methods:**

A decision analytic model using the Markov process was developed covering cardiovascular diseases, type 2 diabetes, and surgical complications. Clinical effectiveness and safety were based on the literature and data from the Scandinavian Obesity Surgery Registry. Gastric bypass, sleeve gastrectomy, and gastric banding were included in the analysis. Cost data were obtained from Swedish sources.

**Results:**

Bariatric surgery was cost saving in comparison with conservative management. It also led to a substantial reduction in lifetime risk of events: from a 16 % reduction in the risk of transient ischaemic attacks to a 62 % reduction in the incidence of type 2 diabetes. Over a lifetime, surgery led to savings of €8408 and generated an additional 0.8 years of life and 4.1 quality-adjusted life years (QALYs) per patient, which translates into gains of 32,390 quality-adjusted person-years and savings of €66 million for the cohort, operated in 2012. Analysis of the consequences of a 3-year delay in surgery provision showed that the overall lifetime cost of treatment may be increased in patients with diabetes or a body mass index >40 kg/m^2^. Delays in surgery may also lead to a loss of clinical benefits: up to 0.6 life years and 1.2 QALYs per patient over a lifetime.

**Conclusion:**

Bariatric surgery, over a lifetime horizon, may lead to significant cost savings to health care systems in addition to the known clinical benefits.

**Electronic supplementary material:**

The online version of this article (doi:10.1007/s11695-014-1567-5) contains supplementary material, which is available to authorized users.

## Introduction/Purpose

Obesity is a global epidemic. Considering the limited effectiveness of conservative weight loss methods in severely obese patients [1–3], bariatric surgery is the only available treatment option. Because of the increased financial pressure, there is an ongoing need to inform decision-makers and surgeons about the economic consequences of bariatric surgery to health care systems in European countries. The objective of the present study was to develop a comprehensive decision analytic model for bariatric surgery to support decision-making for priority setting for the treatment of obesity in European countries.

## Patient Materials and Methods

**Fig. 1:**
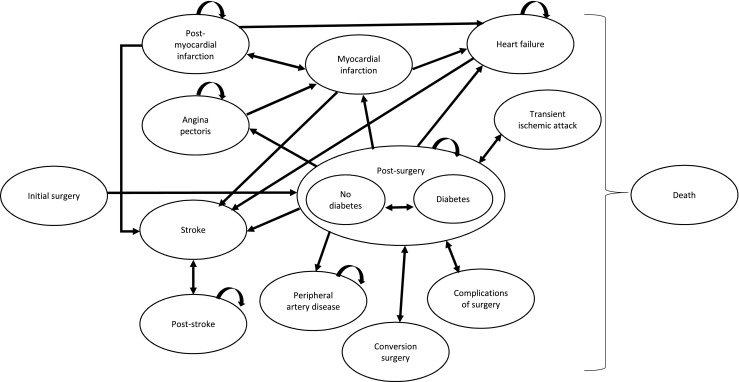
Structure of the Markov model. The figure presents the structure of the Markov model. Patients in the surgical arm enter the model through the “Initial surgery” state and, in the next cycle, move to either “Diabetes post-surgery” or “No Diabetes post-surgery” state depending on the presence or absence of diabetes at the start of the analysis. Patients may recover from diabetes or experience diabetes. From any of the post-surgery state, patients can experience angina pectoris, myocardial infarction, heart failure, transient ischaemic attack, stroke, peripheral arterial disease, complications of surgery, or undergo conversion surgery if weight loss was not achieved. Patients can also move from one negative health state to another (i.e., experience a stroke after being in a heart failure state). Patients may also die from any state. Patients in the medical management arm enter the model either through “Diabetes” or “No diabetes” state. They can experience the same negative events except for complications of surgery or conversion surgery

Decision analytic modeling was employed to evaluate the cost-effectiveness of bariatric surgery. A Markov process [4] was developed covering surgery and post-surgery, post-surgery complications, type 2 diabetes, angina pectoris, myocardial infarction, stroke, transient ischaemic attack, heart failure, and peripheral arterial disease states. In the Markov model during each cycle, which is equal to 1 month, a patient may progress to another health state (e.g., healthy individual in post-surgery state may experience stroke) or remain in the previous state. Each state is associated with specific cost and utility (based on health-related quality of life). The flow of patients in the surgical arm is presented in Fig. 1. The flow of patients in the optimal medical management arm is the same with the exception of absence of initial surgery, conversion surgery, and surgical complications states. Cost-effectiveness was evaluated over a lifetime perspective. Additional information on methods is provided in section S1 of Supplemental Material.

### Input Data

#### Clinical Effectiveness and Safety Data

The model operates by predicting the risk of cardiovascular events, type 2 diabetes, and complications of surgery. The risk of cardiovascular events is predicted by the patient’s characteristics (age, gender, level of systolic blood pressure (SBP), level of body mass index (BMI), presence of diabetes, and smoking status), which can increase or decrease the risk of events [5–9]. Short-term safety was informed by the Michigan Bariatric Surgery Registry data [10, 11], and data for long-term safety of surgery were based on information from the Scandinavian Obesity Surgery Registry (SOREG) [12]. Cholecystectomy, abdominal hernia repair, leakage and abscess, gastric stricture, gastric ulcer, skin surgery, and conversion surgery were considered.

The premise of the analysis is that the risk of cardiovascular events and diabetes depends on multiple risk factors and, by modifying some of these factors (BMI, SBP, presence of diabetes), the overall risk can be modified. By modeling the risk in the surgical arm and in a hypothetical cohort of non-operated patients, it is then possible to quantify the impact of surgery on the rate of long-term adverse events.

Three of the most common surgical approaches were included into the model: gastric bypass (GBP), sleeve gastrectomy (SG), and adjustable gastric banding (GB). Although GB has a very limited utilization is Sweden, it was included for comparative purposes. The relationship between the different surgical methods and the BMI level was derived from SOREG 2011 data for base-case analysis [12]. Using the latest follow-up observation available (2 years), the impact on the BMI was extrapolated using data on BMI change from the Swedish Obese Subjects (SOS) study [13]. After 15 years, the BMI level was assumed stable for the rest of the patient’s life. Changes in BMI in the optimal medical management arm were derived from changes of BMI in the control arm of the SOS study [13]. The major clinical inputs are presented in Table 1 (additional inputs are presented in Table S1).

**Table 1:** Major clinical, cost, and utility inputs

Parameter	Value	Range	Distribution for probabilistic sensitivity analysis	Source
Patient baseline characteristic				
Age, years	41	25–65	Normal (SD = 5)	SOREG 2011 [12]
Gender, males (%)	24	NA	Beta (*α* = 1760; *β* = 5874)	
Body mass index, kg/m2	42.8	30–60	Normal (SE = 5.8)	
Diabetes mellitus, (%)	18.39	NA	Beta (*α* = 1404; *β =* 6230)	
Systolic blood pressure, mmHg	140.1	125–200	Gamma (*α* = 55.53; *λ* = 2.52)	Sjostrom 2004 [9]
Smoking, (%)	14.3	NA	Beta (*α* = 1128; *β* = 6770)	OECD fact book [14]
Absolute BMI reduction, reported in the Scandinavian Obesity Surgery Registry				
GBP, 1-year, males	12.7	8.7–37.7	Normal (SD = 2.2)	SOREG 2011 [12]
GBP, 2-year, males	12.6	8.6–37.4	Normal (SD = 2.2)	
SG, 1-year, males	9.7	5.9–25.5	Normal (SD = 1.7)	
SG, 2-year, males	9.4	5.7–24.7	Normal (SD = 1.6)	
GB, 1-year, males	5.6	3.9–16.9	Normal (SD = 1.0)	
GB, 2-year, males	6.9	4.8–20.9	Normal (SD = 1.2)	
GBP, 1-year, females	13.5	9.5–41.1	Normal (SD = 2.4)	
GBP, 2-year, females	13.5	9.5–41.2	Normal (SD = 2.4)	
SG, 1-year, females	12.5	7.5–32.8	Normal (SD = 2.2)	
SG, 2-year, females	14.7	8.9–38.0	Normal (SD = 2.6)	
GB, 1-year females	5.5	3.9–17.0	Normal (SD = 0.9)	
GB, 2-year, females	5.1	3.6–15.7	Normal (SD = 0.9)	
Other clinical inputs				
Proportion of patients with remission of diabetes at 2 years, surgical arm	0.72	0.67–0.77	Beta (*α* = 246; *β* = 95)	Sjostrom 2004 [9]
Proportion of patients with remission of diabetes at 2 years, OMM arm	0.21	0.15–0.27	Beta (*α* = 52; *β* = 196)	
Proportion of patients with remission of diabetes at 10 years, surgical arm	0.36	0.25–0.50	Beta (*α* = 42; *β* = 76)	
Proportion of patients with remission of diabetes at 10 years, OMM arm	0.13	0.07–0.22	Beta (*α* = 11; *β* = 73)	
Cost inputs, €				
Cost of bariatric surgery without complications	4915	3932–5898	NA	NordDRG tariff L08E
Cost of bariatric surgery with complications	5766	4613–6919	NA	NordDRG tariff L08C
Annual cost of diabetes type 2	2713	1356–5426	Gamma (*α* = 100; *λ* = 305)	Henriksson 2000 [15]
Annual cost of acute stroke	7532	3766–15,063	Gamma (*α* = 100; *λ* = 848)	Ghatnekar 2004 [16]
Annual cost of post-stroke 1 year	7779	3889–15,558	Gamma (*α* = 100; *λ* = 875)	
Annual cost of post-stroke 2 year and onwards	5784	2892–11,569	Gamma (*α* = 100; *λ* = 651)	
Cost of transient ischemic attack	1928	1542–1851	NA	NordDRG DRG tariff A47N
Cost of acute myocardial infarction	4592	2296–9183	Gamma (*α* = 100; *λ* = 516)	Henriksson 2011 [17]
Annual cost of post-MI state	3590	1795–7181	Gamma (*α* = 100; *λ* = 404)	Wilhelmsen 2010 [18]
Annual cost of heart failure	3895	1947–7790	Gamma (*α* = 100; *λ* = 438)	Agvall 2005 [19]
Annual cost of peripheral artery disease	4013	2006–8026	Gamma (*α* = 100; *λ* = 451)	Levy 2003 [20]
Annual cost of angina pectoris	4055	2027–8109	Gamma (*α* = 100; *λ* = 456)	Andersson 1995 [21]

*GB* gastric banding, *GBP* gastric bypass, *MI* myocardial infarction, *OMM* optimal medical management, *SG* sleeve gastrectomy

#### Resource Utilization and Cost Data

Cost data were determined using Swedish sources. The base-case analysis included only direct medical costs.

The number of surgical procedures as well as the rate of use of the different surgical methods (GBP—98 %, SG—1.6 %, GB—0.4 %) were obtained from SOREG [12].

The costs of complications of obesity were obtained from the literature [15–17, 19–21]. The major cost data are presented in Table 1 (additional information in Table S1). For the scenario analysis, the indirect cost of end-stage events was added to the analysis. The cost data are presented in 2012 euros. The inflation adjustment of values in Swedish krona was initially performed using the Swedish consumer price index [22], and values were then, as recommended in the literature [23], converted into euro currency (1 SEK = 0.089 euro) using purchase power parities [24].

#### Utility Data

Health-related quality of life (HRQoL) was expressed on the basis of the generic HRQoL instrument, EuroQol-5D (EQ-5D), and was dependent on the BMI level and the presence of diabetes [25]. The impact of complications of obesity on quality of life was based on the literature [26]. Utility data are presented in Table S1.

#### Cohort Description

Two types of cohorts were evaluated in the model. First, analysis in the so-called multiple cohorts was performed based on a cohort of real candidates for surgery in Sweden. Characteristics of patients for analysis were obtained from SOREG [12], the SOS study [9], and the OECD data [14] (Table 1). Second, the cost-effectiveness of bariatric surgery was estimated in 16 cohorts of 41-year-old non-smoking males and females with different BMI levels: 30–34 (moderate), 35–39 (severe), 40–50 (morbid), and >50 kg/m^2^ (super obese). Further sub-classification was made for the presence or absence of type 2 diabetes.

#### Analysis

The incremental cost-effectiveness ratio (ICER) was calculated by comparing the difference in the average total costs with the difference in the average quality-adjusted life years (QALYs) among the study’s arms. All costs and outcomes beyond the first year were discounted at the rate of 3.0 % annually according to Swedish recommendations [27]. The intervention was considered cost-effective if the ICER was below €35,526 per QALY [28–30]. It means that to be considered cost-effective in Sweden, medical technology needs to lead to additional cost of no more than €35,526 for one extra year of life of full health (QALY).

In addition to the standard evaluation of cost-effectiveness between two treatment options, an analysis was performed on the impact of waiting lists (delay in surgery provision) on the clinical and economic outcomes. The patients were initially included in the optimal medical management arm and then moved to the surgical arm after 3 years. The results were compared with those of patients who underwent immediate surgery.

The model was constructed using Microsoft Excel 2010 (Microsoft Corp., Redmond, WA, USA) and was extensively validated with results provided in Supplementary material.

#### Sensitivity and Scenario Analysis

A one-way deterministic sensitivity analysis was performed to assess the impact of varying the model parameters while holding other variables fixed at base-case values. In addition to a one-way sensitivity analysis, 11 additional scenarios were tested (Section S3). To address sampling uncertainty, a probabilistic sensitivity analysis (PSA) was performed using 5000 Monte Carlo simulations. In PSA, specific distribution (i.e., normal, log-normal, beta, gamma, and uniform) is determined for every parameter; during 5000 runs (simulations) each parameter varies randomly within a pre-specified distribution. The outcomes of interest (cost, life years gained, and QALYs gained) are averaged across 5000 runs.

## Results

### Model Validation

The external validation showed that the model predicts the majority of clinical events (cardiovascular mortality, stroke, health failure, angina, peripheral arterial disease, incidence and remission of diabetes) with a high degree of precision, although there was a tendency to overestimate all-cause mortality and combined (fatal and non-fatal) myocardial infarction. Details of the validation and evaluation of the model’s performance are presented in Section S2.

### Base-Case Results in Multiple Cohorts Extrapolated from SOS and SOREG

In the base-case analysis, bariatric surgery was cost saving in comparison with conservative management. In the simulation, surgery led to substantial reduction in the lifetime risk of negative events (Table 2), from a 16 % reduction in the risk of transient ischaemic attack to a 62 % reduction in the incidence of type 2 diabetes. Over the lifetime of the cohort, surgery led to savings of €8408 and generated an additional 0.8 years of life or 4.1 QALYs per patient (Table 3). In Swedish settings, bariatric surgery becomes cost-effective (i.e., even though surgery may have higher cost, it leads to more benefits, and cost/effect ratio is thus below accepted willingness-to-pay threshold in Sweden) after 2 years (ICER €26,985/QALY) and cost saving (i.e., surgery leads to more benefits at lower cost) after 17 years (Figure S5).

**Table 2:** Number of events and relative risks over lifetime

	Angina	MI total non-fatal	Fatal MI	Stroke total non-fatal	Fatal stroke	TIA	HF	PAD	Diabetes
Absolute risk in surgical arm	11 %	22 %	2 %	18 %	3 %	2 %	15 %	10 %	14 %
Absolute risk in OMM arm	13 %	28 %	3 %	23 %	4 %	2 %	19 %	11 %	36 %
Relative risk	0.82	0.80	0.70	0.79	0.78	0.84	0.84	0.84	0.38

*HF* heart failure, *MI* myocardial infarction, *OMM* optimal medical management, *PAD* peripheral artery disease, *TIA* transient ischemic attack

**Table 3:** Results of cost-effectiveness analysis

	Cost, €	∆ cost, €	LYG, years	∆ LYG	QALYs gained	∆ QALYs	ICER, €/QALY
OMM arm	34,665	−	21.4	−	9.4	−	−
Surgical arm	26,258	−8408	22.2	0.8	13.5	4.1	Dominates

Table presents results of cost-effectiveness analysis. Results demonstrate that surgery leads to lower cost and higher health gains compared with non-surgical management, so surgery dominates over conservative management

*ICER* incremental cost-effectiveness ratio, *LYG* life years gained, *OMM* optimal medical management, *QALYs* quality-adjusted life years

### Results in Specific Cohorts of Patients

Analysis in specific cohorts revealed that bariatric surgery is cost saving in all of the four pre-specified diabetic cohorts (moderately, severely, morbidly, and super obese) in both male and female patients. In the non-diabetic cohorts, surgery was cost saving in all cohorts except for moderately obese male (ICER €459/QALYs) and female (ICER €51/QALYs) patients. In these two cohorts, surgery remained very cost-effective (well below the willingness-to-pay threshold of €35,526/QALYs). Detailed results are provided in Tables S9-12. The degree of clinical benefits for the male and female cohorts is outlined in Fig. S6A-B.

### Impact of Waiting Lists on the Clinical and Economic Outcomes of Bariatric Surgery

The analysis of the consequences of a 3-year delay in providing surgery showed that the overall lifetime cost in the surgical arm may be slightly reduced in non-diabetic patients with moderate and severe obesity (BMI < 40 kg/m^2^), but the cost was increased in non-diabetic patients with morbid or super obesity and diabetic patients (increase from €23 to €2 803) (Table 4, additional data in Table S13). Time delay in surgery led to significant losses of clinical benefits (in the range of 0 and 0.6 for life years and 0.2 and 1.2 for QALYs). Losses of clinical benefits are higher in males and diabetic patients.

**Table 4:** Impact of 3-year delay in surgery provision on total cost of treatment, life years, and QALYs gained in different cohorts of patients

Population	Moderately obese		Severely obese		Morbidly obese		Super obese	
	Males	Females	Males	Females	Males	Females	Males	Females
Difference in total cost, €								
Non-diabetic	−437	−448	−439	−467	26	−6	196	170
Diabetic	2145	2708	2062	2625	2299	2803	2066	2551
Difference in life years gained								
Non-diabetic	−0.1	0	−0.2	−0.1	−0.2	−0.1	−0.2	−0.1
Diabetic	−0.6	−0.1	−0.6	0	−0.6	0	−0.6	−0.1
Difference in quality-adjusted life years gained								
Non-diabetic	−0.3	−0.2	−0.3	−0.3	−0.6	−0.5	−0.8	−0.7
Diabetic	−0.7	−0.4	−0.7	−0.4	−1	−0.6	−1.2	−0.8

Table presents modeled difference in cost and clinical outcomes between delayed and immediate surgery. Negative cost value indicates that delayed surgery leads to reduction of cost compared with immediate surgery. Positive cost value indicates that delayed surgery leads to increased cost compared with immediate surgery. Negative value of life years or QALYs gained indicates that delayed surgery leads to reduction of health benefits. For example, in moderately obese diabetic males, delayed surgery will lead to increase of cost of €2 145 and loss of 0.6 life years or 0.7 QALYs

### Sensitivity Analyses

Deterministic one-way sensitivity analysis showed that four parameters can affect the cost saving effect of surgery (i.e., surgery becomes cost-effective): (1) the magnitude of the effect of surgery, (2) start age (better to operate patients when they are younger), (3) BMI (better to operate patients when BMI is lower), and (4) inclusion of an annual visit to a surgeon during the follow-up program from year three and onwards. Change of cost variables with 50 % variations did not influence the cost saving effect of surgery. The most sensitive parameter from cost variables was the annual cost of type 2 diabetes.

**Fig. 2:**
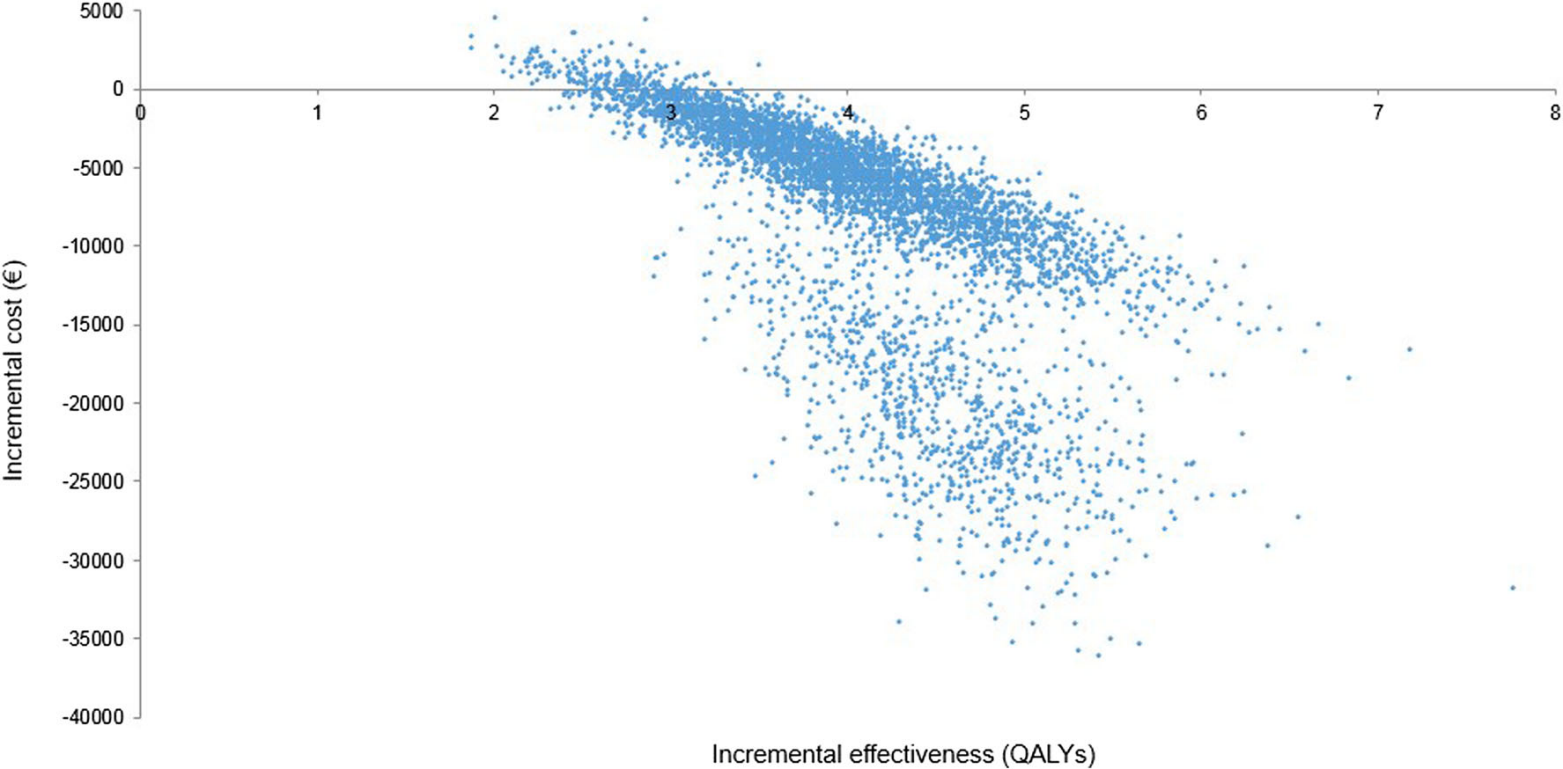
Cost-effectiveness acceptability plane. The figure shows results of probabilistic sensitivity analysis at the lifetime horizon. Each *dot* represents results (change in cost and QALYs) for one simulated patient. The figure presents two populations which differ by the presence or absence of diabetes mellitus at the start of the model (diabetic patients have a higher level of cost saving). Analysis shows that bariatric surgery leads to additional benefits (increase in QALYs) in all patients, and cost saving (lower cost compared with continuation of optimal medical treatment) in majority of patients

The probabilistic sensitivity analysis demonstrated that bariatric surgery produces clinical benefits (additional QALYs) in all patients and has a cost saving effect in 99.1 % of cases while, in the remaining 0.9 %, it is cost-effective (Fig. 2).

Additional 11 scenario analyses showed that uncertainty around the model inputs and structure did not affect the main results significantly (Section S4).

## Discussion

The present study examines economic consequences of bariatric surgery in patients with severe obesity. As health care systems are operating under significant resource constraints, it is important to ensure that health interventions are either aiming to reduce the cost of care or provide good value (clinical benefits) for the money spent (i.e., technologies are cost-effective). Like all other health interventions, bariatric surgery can be evaluated from an economic perspective to support decision-making about the appropriateness of funds allocation for this service.

In the present analysis, a decision analytic technique with Markov modeling was used to evaluate the long-term economic impact of surgery in the context of the Swedish health care system. Decision analytic modeling is a well-established approach in health economics [4, 23, 31] and is recognized by health technology assessment bodies around the world [32–34] and is included into the standard procedures for the assessment of cost-effectiveness of technologies over a long time horizon. Markov modeling was used in the vast majority of the previous decision analytic models in the field of bariatric surgery [7, 25, 35–42].

This cost-effectiveness analysis showed, in Swedish settings, that bariatric surgery has a cost saving effect on the health care system over the patient’s lifetime and is associated with substantial clinical benefits. If these clinical benefits are extrapolated to the entire population of Swedish patients who underwent surgery in 2012 (*n* = 7900 from a population of around nine million people), it would result in a gain of 6320 person-years or 32,390 quality-adjusted person-years. Over the lifetime of the treated cohort, the Swedish health care system will save up to €66 million. Both amounts of cost savings and additional life years/QALYs over lifetime are provided after discounting at 3 % rate annually. This is in line with standard methodology in health economics [4, 23, 31] and Swedish recommendations [27], and it is used to reflect important phenomena of patients valuing immediate benefits more, than benefits in the future. When no discounting is applied, expected benefits and cost saving are even greater (Table S14). The results of the analysis were stable in multiple sensitivity and scenario analyses including usage of very conservative estimate of the effect of surgery on BMI from recent systematic literature review and network meta-analysis [44].

In addition to the ability to save cost to the health care system over the lifetime of the patients, bariatric surgery was shown to be cost-effective already 2 years after procedure. Although decision-makers in Sweden and other European countries usually require long enough time horizon for analysis to capture all clinical and economic consequences of intervention, ability to rapidly demonstrate good value for money can support implementation of surgery among private payers/insurers.

Our analysis is the first attempt to quantify the potential impact of extensive waiting lists on the cost and clinical outcomes of bariatric surgery in Sweden. While it indicates the importance of reducing waiting time, few studies have specifically focused on this parameter [43, 45, 46]. Results of the present study highlight the necessity to reduce waiting lists and to remove unnecessary barriers to allow a greater utilization of surgery for patients unresponsive to conventional medical management.

The analysis also showed that gastric bypass remains the most economically beneficial surgical option. Although gastric bypass is the dominant treatment option in Sweden, we have tested a number of hypothetical scenarios of reduction of use of gastric bypass and corresponding increase of use of sleeve gastrectomy and adjustable gastric banding. Reduction of proportion of gastric bypass from 98 to 60 % will result in a loss of €1156 and 0.6 QALYs per patient and, for the patient population who underwent surgery in 2012, it represents loss of €9.1 million and 2844 QALYs for the lifetime of the cohort.

Our analysis is based on a comprehensive decision analytic model, which had both internal technical and external validation against three large clinical studies and the Scandinavian Obesity Surgery Registry. The present study extends the already existing body of evidence on the economic impact of surgery either derived from decision analysis modeling [7, 25, 35–40, 47–49] or real-world economic analyses [50–57]. Our results are in agreement with the overall estimates from other analyses. These studies have either shown the cost saving effect of surgery or its very high cost-effectiveness, which places bariatric surgery in a preferable position when health care priorities have to be established.

Another interesting finding of the analysis is negligible impact of increase of proportion of high-volume centers on cost of surgery over lifetime of patients (Figures S7 and S8). Although improved quality of care may have important short-term costs and clinical outcomes, over the lifetime of the cohort, it does not play an important role, as key cost drivers are cost of long-term complications of surgery.

The study has a number of limitations. First, we acknowledge that every decision analytic model is a simplification of true health care systems and ideal source of information about cost and clinical benefits of comparative treatment strategies should be derived from randomized controlled trials (RCTs). Nevertheless, in situations where there is a lack of RCTs with appropriate comparators, duration of follow-up, and comprehensive data collection, modeling is inevitable. Second, our model does not include all potential obesity-related diseases (obstructive sleep apnea, musculoskeletal disorders, cancer, obstetrics and gynecology disorders) for which clinical evidence of the beneficial effect of bariatric surgery is emerging. The inclusion of these health states may further increase its cost benefit. Third, our model does not distinguish between the different populations of diabetic patients who may have better or worse outcomes of surgical intervention as reported in a number of recent studies [58–60]. Fourth, the current surgical and conventional management approaches may differ from those used in the studies that provided the major data inputs (i.e., SOS study). Fifth, the utilization of sleeve gastrectomy and gastric banding is very limited in Sweden. Thus, the extrapolation of our results to countries with a higher utilization of these two procedures may be limited. Sixth, data about routine pre- and post-surgery as well as routine conservative management of surgical candidates in Sweden were limited, so assumptions were required. As it was shown in the sensitivity analysis, the cost of routine post-surgery care may influence the cost-effectiveness of bariatric surgery.

In conclusion, using a comprehensive decision analytic model over the patient’s lifetime, we have shown that bariatric surgery is associated with significant clinical benefits that lead to cost savings to the health care system.

## Electronic Supplementary Material

Below is the link to the electronic supplementary material.

ESM 1 (DOCX 315 kb)
