# Supplementary material for: Bariatric Surgery can Lead to Net Cost Savings to Health Care Systems: Results from a Comprehensive European Decision Analytic Model
Source: Obes Surg. 2015 Feb 2;25(9):1559–68. doi: 10.1007/s11695-014-1567-5 (PMC4522026; doi:10.1007/s11695-014-1567-5)
Supplement: Supplementary file 1 — (DOCX 315 kb) [file 11695_2014_1567_MOESM1_ESM.docx]

**Supplemental material for the manuscript**

**Bariatric surgery can lead to net cost savings to health care systems: results from a comprehensive European decision analytic model**

**Section S1.** **Additional Description of Methods**

Methods and results are reported in accordance with the Consolidated Health Economic Evaluation Reporting Standards (CHEERS)^(61)^.

Inputs to the model are presented for base-case (main; with structure, clinical and cost inputs defined as primary ones) analysis and scenario analysis – analysis in which the major elements of the model are changed to evaluate their impact on the results of the analysis.

**Clinical Effectiveness and Safety Data**

The risk equation from the Framingham Heart Study was used to determine the 10-year risk of cardiovascular events, which was re-calculated into monthly risk^(5, 6)^. In the model, cardiovascular risk was re-calculated annually for the initial 8 years (the longest follow-up reported in the included studies) and, subsequently, every 10 years. The incidence of diabetes was BMI-related and determined by polynomial regression as reported by Picot *et al.*^(7)^, and based on estimates from Colditz *et al.*^(8)^. Data on remission of diabetes were obtained from the 2- and 10-year data from the Swedish Obesity Subjects study^(9).^  No new episodes of remission were assumed after 10 years post-surgery.

The risk of short-term (30-day) mortality and serious adverse events in base-case analysis was based on the Michigan Bariatric Surgery Registry data, which allowed analysis of volume-outcome relationships (e.g., lower rate of complications with increased hospital volume)^(10, 11)^. In the scenario analysis, estimates from the Scandinavian Obesity Surgery Registry (SOREG) were used^(12)^. The SOREG 2011 data were also used to estimate the 2-year risk of complications of surgery and the consequences of rapid weight loss (cholecystectomy, abdominal hernia repair, leakage and abscess, gastric stricture, gastric ulcer)^(12)^. Skin surgery due to rapid weight loss was also considered, and data were obtained from SOREG. The rate of conversion surgery was obtained from a controlled study of gastric bypass and adjustable gastric banding over 4.2 and 3.6 years of follow-up, respectively^(50)^. The probability of conversion surgery after sleeve gastrectomy was assumed the same as for gastric bypass.

Gender-specific Swedish life tables provided the mortality rate in the general population. The non-ischaemic heart disease mortality rate in the general population was calculated by subtracting the mortality due to ischaemic heart disease (ICD-10 codes I20-I25) from all-cause mortality. Based on data from numerous epidemiological studies, the presence of one cardiovascular disease state or diabetes influences the risk of having associated conditions (e.g., the risk of stroke is higher in patients who have heart failure) and, consequently, mortality.

For non-diabetic patients, changes in SBP were derived from the SOS study^(9)^ while, for diabetic patients, they were derived from the study of Ikramuddin *et al.*^(62)^. Based on the latest follow-up observation available, the level of SBP was assumed stable for the rest of the patient’s life. For the analysis in different cohorts of patients, the BMI change was retrieved from individual studies^(50, 63-71)^.

The transformation of transition probabilities into monthly probabilities for different time horizons was performed using a standard approach^(31)^.

**Resource Utilization and Cost Data**

The cost of bariatric surgery procedure was set as that of the Swedish national DRG tariff from 2012 (DRG L08E). Cases with serious in-hospital complications were assigned higher DRG tariff (DRG L08C). Post-surgery care was derived from European guidelines^(72)^ and validated by a clinical expert. It was assumed that surgical candidates who did not undergo surgery required an annual visit to a surgeon in 5% of cases. The unit cost of out-patient nurse and physician visits was obtained from the Västra Götaland Regional Health Authority^(73)^.

The number of high- (more than 250 cases annually), medium- (between 100 and 250 cases) and low-volume (less than 100 cases) centres was obtained from the SOREG report^(12)^.

For indirect costs, the loss of productivity from paid and unpaid work due to acute illness and from early retirement was considered, and based on the available literature^(21, 74-76)^. As the data were limited to a 1-year time horizon, they were extrapolated to further years unless the patient reached the age of 65 years, which is the standard retirement age in Sweden.

**Model Validation**

A three-step validation process was employed. First, the face validity of modelling results was assessed. Second, numerous “stress tests” were performed to verify the technical performance of the model (*Table S2*). Third, an external validation of the model was performed using three large epidemiological studies (ASCOT-BPLA^(77)^, AHEAD^(78)^ and ACCORD^(79)^) and the interventional quality registry SOREG^(12)^ (Section S1).

**Sensitivity and Scenario Analysis**

In one-way sensitivity analysis, the cost drivers (variables with a major input to the costs) were identified. For deterministic sensitivity analysis, a single cohort of 41-year old non-smoking males with a BMI of 42.8 kg/m^2^, a SBP of 140.1 mm Hg and no history of diabetes was used.

**Table S1** Additional clinical, cost and utility inputs

| Parameter | Value | Range | Distribution for probabilistic sensitivity analysis | | Source |
| --- | --- | --- | --- | --- | --- |
| Absolute BMI reduction from individual studies (for single cohort analysis) | | | | | |
| GBP, moderately obese with diabetes, 1-year | 9.1 | 1.3-27.1 | Normal (SD=1.6) | Ikramuddin 2013 (63) | |
| GBP, severely obese with diabetes, 1-year | 10.2 | 2.1-28.9 | Normal (SD=1.8) | Schauer 2012 (64) | |
| GBP, morbidly obese, 1-year, best case | 15.9 | 5.5-38.0 | Normal (SD=2.8) | Nguyen 2009 (50) | |
| GBP, morbidly obese, 1-year, worst case | 8.4 | 0-33.1 | Normal (SD=1.5) | Angrisani 2007 (65) | |
| GBP, morbidly obese, 2-year absolute BMI reduction, best case | 16.9 | 7.7-38.3 | Normal (SD=3.0) | Nguyen 2009 (50) | |
| GBP, morbidly obese, 2-year, worst case | 10.7 | 0.8-33.7 | Normal (SD=3.0) | Stoeckli 2004 (66) | |
| GBP, morbidly obese, 3-year, best case | 16.7 | 7.4-46.6 | Normal (SD=3.0) | Nguyen 2009 (50) | |
| GBP, morbidly obese, 3-year, worst case | 14.7 | 5.9-35.0 | Normal (SD=2.6) | Angrisani 2007 (65) | |
| GBP, morbidly obese, 4-year | 17 | 7.8-38.3 | Normal (SD=3.0) | Nguyen 2009 (50) | |
| GBP, morbidly obese, 5-year | 14 | 5.0-34.8 | Normal (SD=2.5) | Angrisani 2007 (65) | |
| GBP, super obese, 1-year | 18.5 | 6.2-47.2 | Normal (SD=3.3) | Bessler 2007 (67) | |
| GBP, super obese, 2-year | 22 | 10.7-48.2 | Normal (SD=3.9) |  |  |
| SG, severely obese with diabetes, 1-year | 9 | 0.9-28.1 | Normal (SD=1.6) | Schauer 2012 (64) | |
| SG, morbidly obese, 1-year | 17.7 | 9.0-37.9 | Normal (SD=3.1) | Karamanakos 2008 (68) | |
| GB, severely obese with diabetes, 1-year | 3.7 | 0-27.0 | Normal (SD=0.6) | Dixon 2008 (69) | |
| GB, severely obese with diabetes, 2-year | 7.5 | 0-28.1 | Normal (SD=1.3) |  |  |
| GB, morbidly obese, 1-year, best case | 11.7 | 1.2-36.2 | Normal (SD=2.1) | Van Dielen 2005 (70) | |
| GB, morbidly obese, 1-year, worst case | 4.7 | 0-31.7 | Normal (SD=0.8) | Angrisani 2007 (65) | |
| GB, morbidly obese, 2-year, best case | 12.1 | 1.7-36.3 | Normal (SD=2.1) | Van Dielen 2005 (70) | |
| GB, morbidly obese, 2-year, worst case | 8.5 | 0-31.7 | Normal (SD=1.5) | Stoeckli 2004 (66) | |
| GB, morbidly obese, 3-year, best case | 11.6 | 0-38.9 | Normal (SD=2.0) | Mathus Vliegen 2007 (71) | |
| GB, morbidly obese, 3-year, worst case | 8.5 | 0-32.7 | Normal (SD=1.5) | Angrisani 2007 (65) | |
| GB, morbidly obese, 4-year, best case | 11.5 | 0-38.9 | Normal (SD=2.0) | Mathus Vliegen 2007 (71) | |
| GB, morbidly obese, 4-year, worst case | 8.15 | 0-31.8 | Normal (SD=1.4) | Angrisani 2007 (65) | |
| GB, morbidly obese, 5-year, best case | 7.3 | 0-38.9 | Normal (SD=1.3) | Mathus Vliegen 2007 (71) | |
| GB, morbidly obese, 5-year, worst case | 8.5 | 0-32.9 | Normal (SD=1.5) | Angrisani 2007 (65) | |
| BMI reduction from Scandinavian Obesity Surgery Registry | | | | | |
| GBP, 1-year, absolute BMI reduction, males, revision | 7.9 | 5.9-25.9 | Normal (SD=1.4) | SOREG 2011 (12) | |
| GBP, 2-year, absolute BMI reduction, males, revision | 7.2 | 5.4-23.6 | Normal (SD=1.2) |  |  |
| GBP, 1-year, absolute BMI reduction, females, revision | 8.8 | 6.7-29.1 | Normal (SD=1.5) |  |  |
| GBP, 2-year, absolute BMI reduction, females, revision | 8.6 | 6.5-28.4 | Normal (SD=1.5) |  |  |
| BMI reduction from network meta-analysis | | | | | |
| GBP, network meta-analysis, 1-year, BMI absolute reduction | 9.0 | 2.7-11.7 | Normal (SD=1.9) | Padwal 2011 (44) | |
| SG, network meta-analysis, 1-year, BMI absolute reduction | 10.1 | 3.0-13.1 | Normal (SD=2.5) |  |  |
| GB, network meta-analysis, 1-year, BMI absolute reduction | 2.4 | 0.7-3.1 | Normal (SD=2.1) |  |  |
| Probability of 30-day mortality and serious complications | | | | | |
| Mortality <100 cases, GBP | 0.01232 | - | - | Michigan Bariatric Surgery Registry (10, 11) | |
| Mortality <100 cases, SG | 0.00748 | - | - |  |  |
| Mortality <100 cases, GB | 0.00286 | - | - |  |  |
| Mortality > 100 and < 249 cases, GBP | 0.00814 | - | - |  |  |
| Mortality > 100 and < 249 cases, SG | 0.00484 | - | - |  |  |
| Mortality > 100 and < 249 cases, GB | 0.00176 | - | - |  |  |
| Mortality > 250, GBP | 0.00682 | - | - |  |  |
| Mortality > 250, SG | 0.00418 | - | - |  |  |
| Mortality > 250, GB | 0.001672 | - | - |  |  |
| Overall serious complications <100 cases, GBP | 0.04368 | - | - |  |  |
| Overall serious complications <100 cases, SG | 0.02652 | - | - |  |  |
| Overall serious complications <100 cases, GB | 0.01014 | - | - |  |  |
| Overall serious complications > 100 and < 249 cases, GBP | 0.02886 | - | - |  |  |
| Overall serious complications > 100 and < 249 cases, SG | 0.01716 | - | - |  |  |
| Overall serious complications > 100 and < 249 cases, GB | 0.00624 | - | - |  |  |
| Overall serious complications > 250, GBP | 0.02418 | - | - |  |  |
| Overall serious complications > 250, SG | 0.01482 | - | - |  |  |
| Overall serious complications > 250, GB | 0.005928 | - | - |  |  |
| 30-day mortality | 0.00024 | 0.000072-0.00041 | Beta (α=1; β=4029) | SOREG 2011 (12) | |
| 30-day serious complications with reoperations | 0.0637 | 0.019-0.11 | Beta (α=257; β=3773) |  |  |
| Annual probability of long-term serious adverse events | | | | | |
| Cholecystectomy, 1-year, GBP, males | 0.007 | 0.0021-0.0119 | Beta (α=23; β=3217) | SOREG 2011 (12) | |
| Cholecystectomy, 1-year, GBP, females | 0.018 | 0.0054-0.0306 | Beta (α=188; β=10266) |  |  |
| Cholecystectomy, 1-year, SG | 0 | NA | Beta (α=0; β=46) |  |  |
| Cholecystectomy, 1-year, GB | 0.01 | 0.003-0.017 | Beta (α=2; β=191) |  |  |
| Cholecystectomy, 2-years, GBP, males | 0.01 | 0.003-0.017 | Beta (α=44; β=4381) |  |  |
| Cholecystectomy, 2-years, GBP, females | 0.022 | 0.0066-0.0374 | Beta (α=29; β=1311) |  |  |
| Cholecystectomy, 2-years, SG | 0 | NA | Beta (α=0; β=16) |  |  |
| Cholecystectomy, 2-years, GB | 0 | NA | Beta (α=0; β=121) |  |  |
| Abdominal wall hernia operations, 1-year, GBP, males | 0.008 | 0.0024-0.0136 | Beta (α=28; β=3423) |  |  |
| Abdominal wall hernia operations, 1-year, GBP, females | 0.009 | 0.0027-0.0153 | Beta (α=100; β=10955) |  |  |
| Abdominal wall hernia operations, 1-year, SG | 0.019 | 0.0057-0.0323 | Beta (α=1; β=51) |  |  |
| Abdominal wall hernia operations, 1-year, GB | 0.005 | 0.0015-0.0085 | Beta (α=1; β=197) |  |  |
| Abdominal wall hernia operations, 2-year, GBP, males | 0.013 | 0.0039-0.0221 | Beta (α=19; β=1453) |  |  |
| Abdominal wall hernia operations, 2-year, GBP, females | 0.012 | 0.0036-0.0204 | Beta (α=58; β=4733) |  |  |
| Abdominal wall hernia operations, 2-year, SG | 0 | NA | Beta (α=0; β=19) |  |  |
| Abdominal wall hernia operations, 2-year, GB | 0.031 | 0.0093-0.0527 | Beta (α=4; β=127) |  |  |
| Banding operations, 1-year, | 0.036 | 0.0108-0.0612 | Beta (α=7; β=191) |  |  |
| Banding operations , 2-year, | 0.07 | 0.021-0.119 | Beta (α=9; β=122) |  |  |
| Plastic operations, 1-year, GBP, males | 0.001 | 0.0003-0.0017 | Beta (α=3; β=3237) |  |  |
| Plastic operations, 1-year, GBP, females | 0.005 | 0.0015-0.0085 | Beta (α=52; β=10454) |  |  |
| Plastic operations, 1-year, SG | 0 | NA | Beta (α=0; β=46) |  |  |
| Plastic operations, 1-year, GB | 0 | NA | Beta (α=0; β=193) |  |  |
| Plastic operations, 2-year, GBP, males | 0.021 | 0.0063-0.0357 | Beta (α=28; β=1312) |  |  |
| Plastic operations, 2-year, GBP, females | 0.059 | 0.0177-0.1003 | Beta (α=261; β=4164) |  |  |
| Plastic operations, 2-year, SG | 0.063 | 0.0189-0.1071 | Beta (α=1; β=15) |  |  |
| Plastic operations, 2-year, GB | 0.008 | 0.0024-0.0136 | Beta (α=1; β=120) |  |  |
| Leakage and abscess, 1-year, male | 0.005 | 0.0015-0.0085 | NA |  |  |
| Leakage and abscess, 1-year, female | 0.002 | 0.0006-0.0034 | NA |  |  |
| Leakage and abscess, 2-year, male | 0.001 | 0.0003-0.0017 | NA |  |  |
| Leakage and abscess, 2-year, female | 0.001 | 0.0003-0.0017 | NA |  |  |
| Obstruction, 1-year, male | 0.019 | 0.0057-0.032 | NA |  |  |
| Obstruction, 1-year, female | 0.018 | 0.0054-0.03 | NA |  |  |
| Obstruction, 2-year, male | 0.021 | 0.0063-0.036 | NA |  |  |
| Obstruction, 2-year, female | 0.03 | 0.009-0.051 | NA |  |  |
| Stricture, 1-year, male | 0.003 | 0.0009-0.0051 | NA |  |  |
| Stricture, 1-year, female | 0.002 | 0.0006-0.0034 | NA |  |  |
| Stricture, 2-year, male | 0.002 | 0.0006-0.0034 | NA |  |  |
| Stricture, 2-year, female | 0.001 | 0.0003-0.0017 | NA |  |  |
| Gastric ulcer, 1-year, male | 0.016 | 0.0048-0.027 | NA |  |  |
| Gastric ulcer, 1-year, female | 0.011 | 0.0033-0.018 | NA |  |  |
| Gastric ulcer , 2-year, male | 0.013 | 0.0039-0.022 | NA |  |  |
| Gastric ulcer , 2-year, female | 0.009 | 0.0027-0.015 | NA |  |  |
| Cholecystectomy 1-year, revision surgery | 0.014 | 0.0042-0.0238 | Beta (α=10; β=688) |  |  |
| Cholecystectomy 2-year, revision surgery | 0.007 | 0.0021-0.0119 | Beta (α=3; β=408) |  |  |
| Hernia operations 1-year, revision surgery | 0.056 | 0.0168-0.095 | Beta (α=42; β=700) |  |  |
| Hernia operations 2-year, revision surgery | 0.043 | 0.0129-0.073 | Beta (α=19; β=424) |  |  |
| Other complications 1-year, revision surgery | 0.074 | 0.0222-0.12 | Beta (α=55; β=687) |  |  |
| Other complications 2-year, revision surgery | 0.055 | 0.0165-0.093 | Beta (α=24; β=419) |  |  |
| Plastic operations 1-year, revision surgery | 0.013 | 0.0039-0.022 | Beta (α=9; β=689) |  |  |
| Conversion from GBP or SG (4.2-year follow-up) | 0.009 | 0.0027-0.0153 | Beta (α=1; β=110) | Nguyen 2009 (50) | |
| Conversion from GB (3.6-year follow-up) | 0.058 | 0.0174-0.0986 | Beta (α=5; β=81) |  |  |
| Other clinical inputs | | | | | |
| Proportion of non-fatal stroke, males | 0.87 | 0.076-0.957 | Beta (α=41; β=6) | Wolf 1992 (80) | |
| Proportion of non-fatal stroke, females | 0.82 | 0.067-0.902 | Beta (α=33; β=7) |  |  |
| Relative risk of death in post-stroke condition | 2.3 | 0.529-2.53 | Log-normal (SE_log_=0.25) | Dennis 1993 (81) | |
| Probability of stroke at 30-day after myocardial infarction | 0.0122 | 0.000015-0.013 | Beta (α=172.29; β=14025.38) | Witt 2006 (82) | |
| Probability of stroke at 1 year after myocardial infarction | 0.0214 | 0.00004-0.023 | NA |  |  |
| Probability of stroke during 1st year after heart failure | 0.0184 | 0.000034-0.020 | Beta (α=565.39; β=30211.98) |  |  |
| Risk of recurrent stroke (2.5 year follow-up) | 0.087 | 0.00076-0.096 | Beta (α=413.04; β=4343.54) | Ovbiagele 2011 (83) | |
| Acute mortality for recurrent stroke | 0.19 | 0.0036-0.209 | Beta (α=129; β=546) | Dennis 1993 (81) | |
| Relative risk of all-cause mortality in post-myocardial infarction state | 3.2 | 1.024-3.52 | Log-normal (SE_log_=0.09) | Rosengren 1998 (84) | |
| Relative risk of recurrent myocardial infarction | 1.78 | 0.32-1.958 | Log-normal (SE_log_=0.12) | Zanchetti 2001 (85) | |
| Acute mortality for recurrent myocardial infarction | 0.161 | NA | Beta (α=4.28; β=22.29) | Krumholz 2009 (86) | |
| Relative risk of death in heart failure state | 4.01 | 1.61-4.411 | Log-normal (SE_log_=0.082) | Arnold 2003 (87) | |
| Probability of HF within 30 days after myocardial infarction | 0.231 | 0.0053-0.25 | Beta (α=45; β=150) | Velagaleti 2008 (88) | |
| 5-year probability of heart failure in post-myocardial infarction state | 0.148 | 0.0022-0.16 | Beta (α=21; β=121) |  |  |
| Relative risk of all-cause death from angina state | 1.63 | 0.26-1.79 | Log-normal (SE_log_=0.086) | Rosengren 1998 (84) | |
| Annual risk of MI from angina state | 0.019 | 0.000036-0.021 | Beta (α=73; β=3767) | Poole-Wilson 2004 (89) | |
| Relative risk of all-cause mortality from peripheral artery disease | 3.1 | 0.961-3.41 | Log-normal (SE_log_=0.24) | Criqui 1992 (90) | |
| Relative risk of all-cause mortality from diabetes state at 40-44 years old, male | 17.7 | 31.33-19.47 | - | Hansen 2009 (91) | |
| Relative risk of all-cause mortality from diabetes state at 45-49 years old, male | 2.4 | 0.57-2.64 | - |  |  |
| Relative risk of all-cause mortality from diabetes state at 50-54 years old, male | 2.6 | 0.67-2.86 | - |  |  |
| Relative risk of all-cause mortality from diabetes state at 55-59 years old, male | 2.5 | 0.62-2.75 | - |  |  |
| Relative risk of all-cause mortality from diabetes state at 60-64 years old, male | 2.8 | 0.7843.08 | - |  |  |
| Relative risk of all-cause mortality from diabetes state at 65-69 years old, male | 2.4 | 0.57-2.64 | - |  |  |
| Relative risk of all-cause mortality from diabetes state at 70-74 years old, male | 1.8 | 0.324-1.98 | - |  |  |
| Relative risk of all-cause mortality from diabetes state at 75-79 years old, male | 1.5 | 0.225-1.65 | - |  |  |
| Relative risk of all-cause mortality from diabetes state at 80-85 years old, male | 1.8 | 0.324-1.98 | - |  |  |
| Relative risk of all-cause mortality from diabetes state at 40-44 years old, female | 0 | 0-0 | - |  |  |
| Relative risk of all-cause mortality from diabetes state at 45-49 years old, female | 0 | 0-0 | - |  |  |
| Relative risk of all-cause mortality from diabetes state at 50-54 years old, female | 2.5 | 0.625-2.75 | - |  |  |
| Relative risk of all-cause mortality from diabetes state at 55-59 years old, female | 2.5 | 0.625-2.75 | - |  |  |
| Relative risk of all-cause mortality from diabetes state at 60-64 years old, female | 1.7 | 0.289-1.87 | - |  |  |
| Relative risk of all-cause mortality from diabetes state at 65-69 years old, female | 1.4 | 0.196-1.54 | - |  |  |
| Relative risk of all-cause mortality from diabetes state at 70-74 years old, female | 1.4 | 0.196-1.54 | - |  |  |
| Relative risk of all-cause mortality from diabetes state at 75-79 years old, female | 1.5 | 0.225-1.65 | - |  |  |
| Relative risk of all-cause mortality from diabetes state at 80-85 years old, female | 1.4 | 0.196-1.54 | - |  |  |
| Cost inputs | | | | | |
| Cost of abdominal hernia procedure | 3 799 | 3 039-4 559 | NA | NordDRG tariff F20E | |
| Cost of cholecystectomy | 3 702 | 2 962-4 442 | NA | NordDRG tariff G12E | |
| Cost of abdomen skin/plastic surgery | 2 604 | 2 083-3 125 | NA | Weighted average of 3 DRG tariffs (L50E, L50C, L50A) | |
| Cost of leakage and abscess | 5 011 | 4 009-6 013 | NA | Weighted average of 3 DRG tariffs (F35E, F35C, F35A) | |
| Cost of obstruction | 5 011 | 4 009-6 013 | NA | Weighted average of 3 DRG tariffs (F35E, F35C, F35A) | |
| Cost of stricture | 1 564 | 1 251-1 877 | NA | DRG tariff JDA55 | |
| Cost of gastric ulcer | 37 | 29-44 | NA | 8-week course of 40 mg omeprazole. Cost obtained from TLV database, 2013 | |
| Cost of visit to general practitioner | 121 | 97-145 | NA | Tariffs for 2012 in Västra Götaland Health Region (73) | |
| Cost of visit to nurse | 47 | 37-56 | NA |  |  |
| Cost of visit to dietician | 107 | 86-129 | NA |  |  |
| Cost of visit to surgeon | 226 | 180-271 | NA |  |  |
| Cost of visit to psychologist | 92 | 74-110 | NA |  |  |
| Annual indirect cost of diabetes II type | 2 118 | 1 059-6 354 | Gamma (α=100; λ=238) | Bolin 2009 (74) | |
| Annual indirect cost of stroke 1 year after event | 11 783 | 6 892-35 350 | Gamma (α=100; λ=1326) | Lindgren 2008 (75) | |
| Annual indirect cost of myocardial infarction 1 year after event | 11 503 | 5 751-34 508 | Gamma (α=100; λ=1295) | Zethraeus 1999 (76) | |
| Annual indirect cost of heart failure | 2 669 | 1 333-8 000 | Gamma (α=100; λ=300) |  |  |
| Annual indirect cost of (stable) angina | 8 147 | 4 074-24 442 | Gamma (α=100; λ=917) | Andersson 1995 (21) | |
| Resource use for pre-, post-surgery and routine management of obesity | | | | | |
| Preoperative surgeon visit, surgical arm | 2 | 1-3 | NA | Expert assumption | |
| Preoperative dietician consultation, surgical arm | 1 | 0-2 | NA |  |  |
| Post-discharge nurse visit, surgical arm, first month | 2 | 1-2 | NA |  |  |
| Follow-up dietician contact, 1 year, surgical arm | 1.7 | 0.7-2.7 | NA |  |  |
| Follow-up psychologist visit, 1 year, surgical arm | 0.01 | 0-1.01 | NA |  |  |
| Follow-up surgeon visit, 1 year, surgical arm | 1.7 | 0.7-2.7 | NA |  |  |
| Follow-up nurse visit, 2 year, surgical arm | 0.6 | 0-1.6 | NA |  |  |
| Annual visit to surgeon in OMM arm | 0.05 | 0-1.05 | NA |  |  |
| Utility inputs | | | | | |
| Angina pectoris | -0.0854 | -0.10; -0.068 | Beta (α=396.93; β=37.06) | ICD-9 413 Angina Pectoris. (26) | |
| Acute myocardial infarction | -0.0626 | -0.07; -0.05 | Beta (α=314.76; β=21.02) | ICD-9 410 Acute Myocardial Infarct (26) | |
| Post-myocardial infarction state | -0.0368 | -0.04; -0.03 | Beta (α=50.73; β=1.94) | Sullivan 2011. ICD-9 412 Old Myocardial Infarct (26) | |
| Acute stroke | -0.1171 | -0.14; -0.09 | Beta (α=622.58; β=82.57) | Sullivan 2011. ICD-9 436 Cva (26) | |
| Post-stroke state | -0.0349 | -0.04; -0.03 | Beta (α=70.68; β=2.56) | Sullivan 2011. ICD-9 433 Precerebral Occlusion (26) | |
| Transient ischemic attack | -0.033 | -0.04; -0.03 | Beta (α=61.08; β=2.08) | Sullivan 2011. ICD-9 435 Transient Cereb Ischemia (26) | |
| Peripheral artery disease | -0.0409 | -0.05; -0.03 | Beta (α=75.38; β=3.21) | Sullivan 2011. ICD-9 447 Other Arterial Disease (26) | |
| Heart failure | -0.1167 | -0.14; -0.09 | Beta (α=438.21; β=57.89) | Sullivan 2011. ICD-9 428 Heart Failure (26) | |

DMII: diabetes type 2; GB: gastric banding; GBP: gastric bypass; OMM: optimal medical management; SG: sleeve gastrectomy.

**Table S2** ‘Stress tests’ for model validation

| # | Test | Expected effect | Observed effect | Action required | Results | Repeated observation |
| --- | --- | --- | --- | --- | --- | --- |
| 1 | Plotting graph with lifetime non-discounted cost and graph with lifetime absolute risk of negative events | In visual inspection, difference in cost between arms should correspond to difference in risk of events | 3.5-4 time higher lifetime cost stroke, TIA and post-stroke condition in OMM arm | Inspection of the code for cost of stroke calculation in OMM arm | Bug in the code was found and corrected | In visual inspection, difference in cost between arms correspond to difference in risk of events |
| 2 | Plotting graph with lifetime total (direct and indirect) discounted and non-discounted cost | In visual inspection, non-discounted costs should be higher than discounted costs, except costs, originated during the first year | In visual inspection, non-discounted costs should be higher than discounted costs, except costs, originated during the first year (primary surgery | None | - | - |
| 3 | Comparing of mean lifetime direct and indirect costs, obtained from simulation page by summing up individual costs, and from analysis of individual costs | Costs, obtained with different calculations should be the same | Costs were different, with higher cost, obtained from simulation page | Inspection of the code for total cost estimation | Bug in the code was found and corrected | Costs, obtained with different calculations are equal |
| 4 | Check correspondence of distribution of surgeries and diabetic population with different inputs (change of data inputs, change diabetes status from “No” to “Yes” | Distribution of surgeries and diabetes cohorts should correlate to given scenarios | Distribution of surgeries and diabetes cohorts correlate to given scenarios | None | - | - |
| 5 | Set initial number of patients to 0 | Costs and QALYs equal 0 across treatments | Costs equal 0 across treatments and presents errors (due to dividing by 0) | None | - | - |
| 6 | Set initial number of cases to 1 | ICER unaltered | ICER unaltered | None | - | - |
| 7 | Set unit costs of treatments to 0 | Total cost of treatment = 0 | Total cost of treatment = 0 | None | - | - |
| 8 | Doubled unit costs of treatment | Treatment costs doubled | Costs of treatment doubled | None | - | - |
| 9 | Set treatment to ‘comparator’ and comparator to ‘treatment’ | QALYs and risk ratios to be the same as base-case, but inverted. | Risk ratios and LYG were correct. Utility levels were not and gave wrong QALY results | Needs revisit |  |  |
| 10 | Set mortality rate to 0% | No deaths in model | No deaths in model | None | - | - |
| 11 | Set mortality rate to 100% at all ages | All patients dead at cycle 1 | All patients dead at cycle 1 | None | - | - |
| 12 | Set mortality rate to 100% at age X | All patients dead after x years (starting age 70 - x) but still generate expected costs and QALYs | All patients dead after x years (starting age 70 - x) but still generate expected costs and QALYs | None | - | - |
| 13 | Increase mortality rate | Reduced costs. | Reduced costs. | None | - | - |
| 14 | Health state utilities are 1 for all states | QALY gained equals LYG | QALY gained equals LYG in surgery but not in OMM | Inspection of the code in OMM arm revealed missing cell for Diabetes Type 2 | Bug was corrected | QALY gained equals LYG |
| 15 | Health state utilities and adverse events all set to 0. | Total QALYs = 0 for treatment and comparator. | Total QALYs = 0 for treatment and comparator. | None | - | - |
| 16 | Health state utilities for states all set to 1 and adverse events all set to 0 | Total QALYs same as life years | Total QALYs same as life years | None | - | - |
| 17 | Run one-way sensitivity analysis for all comparisons | Only cost variables relevant to the comparison should be sensitive in the results of analysis and appear in Tornado diagram | Only cost variables relevant to the comparison were sensitive in the results of analysis and appear in Tornado diagram | None | - | - |
| 18 | Set discount rate for cost to 0 | Discounted and non-discounted mean lifetime costs should be equal | Discounted and non-discounted mean lifetime costs are equal | None | - | - |
| 19 | Set start age of cohort at 66 years | Discounted and non-discounted mean lifetime costs should be equal (retirement age in Sweden is 65 years) | Discounted and non-discounted mean lifetime costs are equal (retirement age in Sweden is 65 years) | None | - | - |
| 20 | Change all short-term complication rate to 0 | Cost of primary surgery should be equal to multiplication of number of operations by cost of operation | Cost of primary surgery is equal to multiplication of number of operations by cost of operation | None | - | - |

ICER: incremental cost-effectiveness ratio; LYG: life years gained; OMM: optimal medical management; QALY: quality-adjusted life years.

**Section S2** Details of external model validation

Validation of model outcomes was performed against three large epidemiological studies (ASCOT-BPLA, AHEAD, ACCORD) and SOREG. Details are provided below.

**Validation against ASCOT-BPLA**

The Anglo-Scandinavian Cardiac Outcomes Trial-Blood Pressure Lowering Arm (ASCOT-BPLA) is a multicenter, prospective, randomized controlled trial that included 19 257 hypertensive patients aged 40 to 79 years old and with, at least, three other cardiovascular risk factors. The goal of the study was to compare the effect of non-fatal myocardial infarction and fatal coronary heart disease of combinations of atenolol with a thiazide versus amlopidine with perindopril.

Patients in this study were randomized in two groups: Amlodipine-based and Atenolol based regimes. The Atenolol-based regime group was chosen for validation. As in the ASCOT-BPLA change of SBP is only reported at 5.5 years and change of SBP during the whole study is reported only in a graph, it was assumed that atenolol lead to decrease of SBP of 5 mm Hg at 6-month follow-up, 10 mm Hg – at 12 months, 15 mm Hg – at 24 months, 20 mm Hg – at 36 months, and 25 mm Hg (value, reported in the study) – at 66-month follow-up.

**Table S3** Study patient characteristics (Atenolol-based regime group)

|  | Baseline | 6 months | 12 months | 24 months | 36 months | 5.5 year  Mean (SD) |
| --- | --- | --- | --- | --- | --- | --- |
| Age | 63.0 | - | - | - | - | - |
| Males, % | 77% | - | - | - | - | - |
| BMI, mean (SD) | 28.7 (4.5) | - | - | - | - | - |
| SBP, mean (SD) | 163.9 (18.1) | -5 | -10 | -15 | -20 | -26.2 (17.9) |
| Smoking, % | 32% | - | - | - | - | - |
| Diabetics, % | 27% | - | - | - | - | - |

BMI: body mass index; SBP: systolic blood pressure; SD: standard deviation.

Validation showed, that model predicts clinical events (cardiovascular mortality, fatal and non-fatal stroke, stable and unstable angina, PAD) relatively precisely. There was a tendency to overestimate all-cause mortality, combined outcome of cardiovascular death, MI and stroke, and combined outcome of fatal and non-fatal MI.

**Table S4** Results of model validation against ASCOT-BPLA (median follow-up of 5.5 years)

| Variable | 95% Credible interval | | Mean, model | Median, model | Mean, study |
| --- | --- | --- | --- | --- | --- |
|  | 2.5% | 97.5% |  |  |  |
| All-cause mortality | 2.4% | 44.6% | 14.38% | 11.55% | 9% |
| Cardiovascular mortality | 0.86% | 8.31% | 4.37% | 4.37% | 4% |
| Fatal and non-fatal stroke | 1.72% | 6.33% | 3.89% | 3.78% | 4% |
| Stable and unstable angina | 1.04% | 5.74% | 2.55% | 2.33% | 3% |
| PAD | 1.37% | 6.19% | 3.75% | 3.73% | 2% |
| Development of Diabetes | 0.036% | 4.73% | 1.18% | 0.66% | 8% |
| Cardiovascular death + MI + stroke | 5.07% | 32.13% | 17.52% | 17.10% | 10% |
| Fatal and non-fatal MI | 2.98% | 26.27% | 13.63% | 13.36% | 5% |

PAD: peripheral artery disease; MI: myocardial infarction.

Results are also presented in the Figure S1.


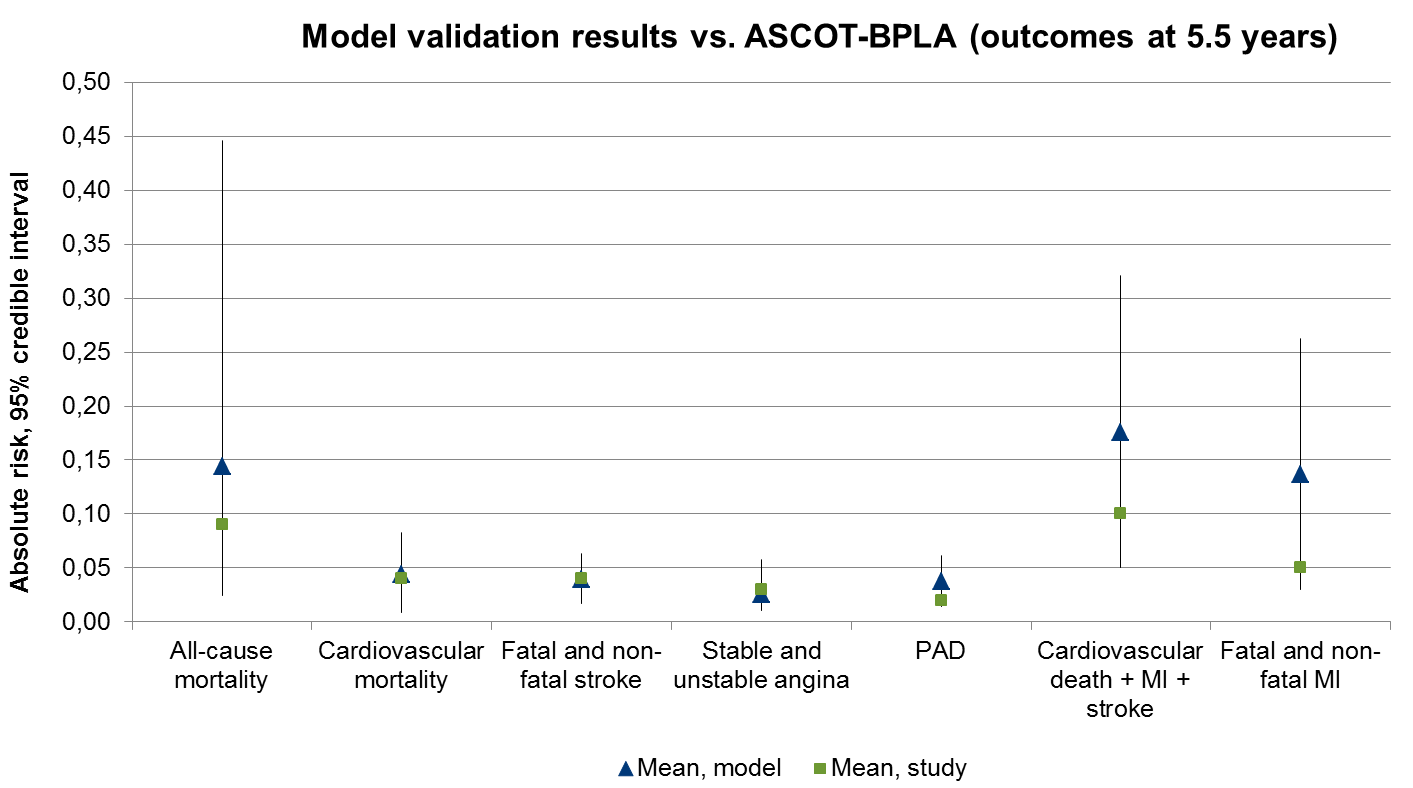


**Fig. S1** Results of model validation against ASCOT-BPLA

**Validation against AHEAD**

The Action for Health in Diabetes (AHEAD) study is a multicenter, prospective, randomized controlled trial that included 5 145 overweight or obese patients with diabetes II. The goal of the study was to compare the effect of an intensive lifestyle intervention that promoted weight loss through decreased caloric intake and increased physical activity (intervention group) or to receive diabetes support and education (control group).

As in the AHEAD change of weight is reported yearly only in a graph, it was assumed that surgery lead to decrease of weight of 8.9% -at 1-year follow-up, 6.9% – at 2 years, 5.9% – at 3 years, 5.44% – at 4 years, 4.9% – at 5 years, 5.44% – at 6 years, 5.44% – at 7 years, 6.43% – at 8 years, 6.9% – at 9 years, and 7.9% at -10 years. Change of weight reported was taken from control group.

**Table S5** Study patient characteristics

|  | Baseline | 1 year | 2 years | 3 years | 4 years | 5 years | 6 years | 7 years | 8 years | 9 years | 10 years |
| --- | --- | --- | --- | --- | --- | --- | --- | --- | --- | --- | --- |
| Age (SD) | 58.6 (6.8) | - | - | - | - | - | - | - | - | - | - |
| Males, % | 40.6% | - | - | - | - | - | - | - | - | - | - |
| BMI, mean (SD) | 35.9 (6.0) | -3.19 | -2.47 | -2.11 | -1.95 | -1.76 | -1.95 | -1.95 | -2.30 | -2.47 | -2.83 |
| SBP, mean (SD) | 128 (17) | - | - | - | - | - | - | - | - | - | - |
| Smoking, % | 4.6% | - | - | - | - | - | - | - | - | - | - |
| Diabetics, % | 100% | - | - | - | - | - | - | - | - | - | - |

BMI: body mass index; SBP: systolic blood pressure; SD: standard deviation.

Validation showed, that model predicts clinical events (cardiovascular mortality, stroke and heart failure) relatively precisely. There was a tendency to overestimate all-cause mortality and combined outcome of fatal and non-fatal MI.

**Table S6** Results of model validation against AHEAD

| Variable | 95% Credible interval | | Mean, model | Median, model | 9.6 years  Mean, study |
| --- | --- | --- | --- | --- | --- |
|  | 2.5% | 97.5% |  |  |  |
| All-cause mortality | 9.32% | 26.09% | 16.13% | 11.87% | 6.77% |
| Cardiovascular mortality | 1.18% | 9.07% | 4.26% | 3.12% | 2.02% |
| Fatal and non-fatal myocardial infarction | 3.41% | 20.84% | 10.30% | 8.74% | 6.34% |
| Stroke | 0.33% | 1.59%% | 0.82% | 0.75% | 3.31% |
| Non-fatal Heart failure | 2.33% | 10.58% | 5.90% | 5.6% | 3.85% |


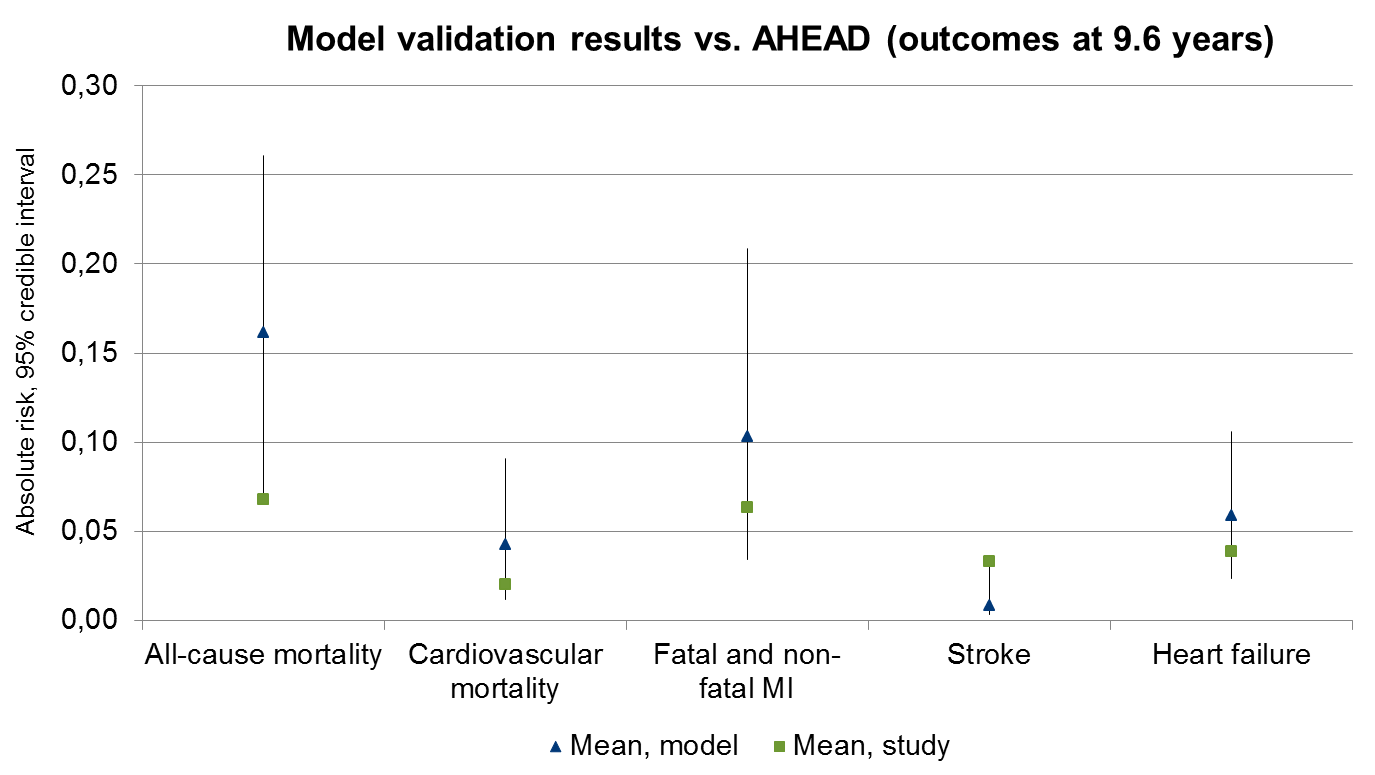


**Fig. S2** Results of model validation against AHEAD

**Validation against ACCORD**

The Action to Control Cardiovascular Risk in Diabetes (ACCORD) study is a multicenter, prospective, randomized controlled trial that included 4 733 patients with diabetes II aged 40 years of age or older with cardiovascular disease or 55 years of age or older with at least two additional risk factors for cardiovascular disease. The goal of the study was to compare the effect of an intensive therapy, targeting a systolic pressure of less than 120 mm Hg, or standard therapy, targeting a systolic pressure of less than 140 mm Hg. The primary composite outcome was nonfatal myocardial infarction, nonfatal stroke, or death from cardiovascular causes.

Results for intensive and standard blood-pressure control in type 2 diabetes patients were reported in a graph only, during an 8-year period. It was assumed that intensive control lead to a 123 mm Hg SBP at 6 months, 122 mm Hg SBP at 9 months, 120 mm Hg SBP at 1 year, 119 mm Hg SBP at 2 years, 119 mm Hg SBP at 3 years, 119 mm Hg SBP at 4 years, 119 mm Hg SBP at 5 years, 119.5 mm Hg SBP at 6 years, 118 mm Hg SBP at 7 years, 120 mm Hg SBP at 8 years.

**Table S7** Study patient characteristics

|  | Baseline | 6 months | 9 months | 1 year | 2 years | 3 years | 4 years | 5 years | 6 years | 7 years | 8 years |
| --- | --- | --- | --- | --- | --- | --- | --- | --- | --- | --- | --- |
| Age (SD) | 62.2 (6.9) | - | - | - | - | - | - | - | - | - | - |
| Males, % | 52.3 | - | - | - | - | - | - | - | - | - | - |
| BMI, mean (SD) | 32.1 (5.6) | - | - | - | - | - | - | - | - | - | - |
| SBP, mean (SD) | 139.2 (15.8) | -16.2 | -17.2 | -19.2 | -20.2 | -20.2 | -20.2 | -20.2 | -19.7 | -21.2 | -19.2 |
| Currently smoking, % | 13.2% | - | - | - | - | - | - | - | - | - | - |
| Diabetics, % | 100% | - | - | - | - | - | - | - | - | - | - |

BMI: body mass index; SBP: systolic blood pressure; SD: standard deviation.

Validation showed, that model predicts clinical events (cardiovascular mortality, stroke and non-fatal MI) relatively precisely. There was a tendency to overestimate all-cause mortality.

**Table S8** Results of model validation against ACCORD

| Variable | 95% Credible interval | | Mean, model | Median, model | 4.7 years  Mean, study |
| --- | --- | --- | --- | --- | --- |
|  | 2.5% | 97.5% |  |  |  |
| All-cause mortality | 5.23% | 15.29% | 9.59% | 12.65% | 6.35% |
| Cardiovascular mortality | 0.93% | 5.85% | 2.65% | 3.54% | 2.54% |
| non-fatal myocardial infarction | 3.03% | 13.04% | 6.10% | 7.78% | 5.33% |
| Fatal or non-fatal Stroke | 2.50% | 4.42% | 2.93% | 2.75% | 1.52% |


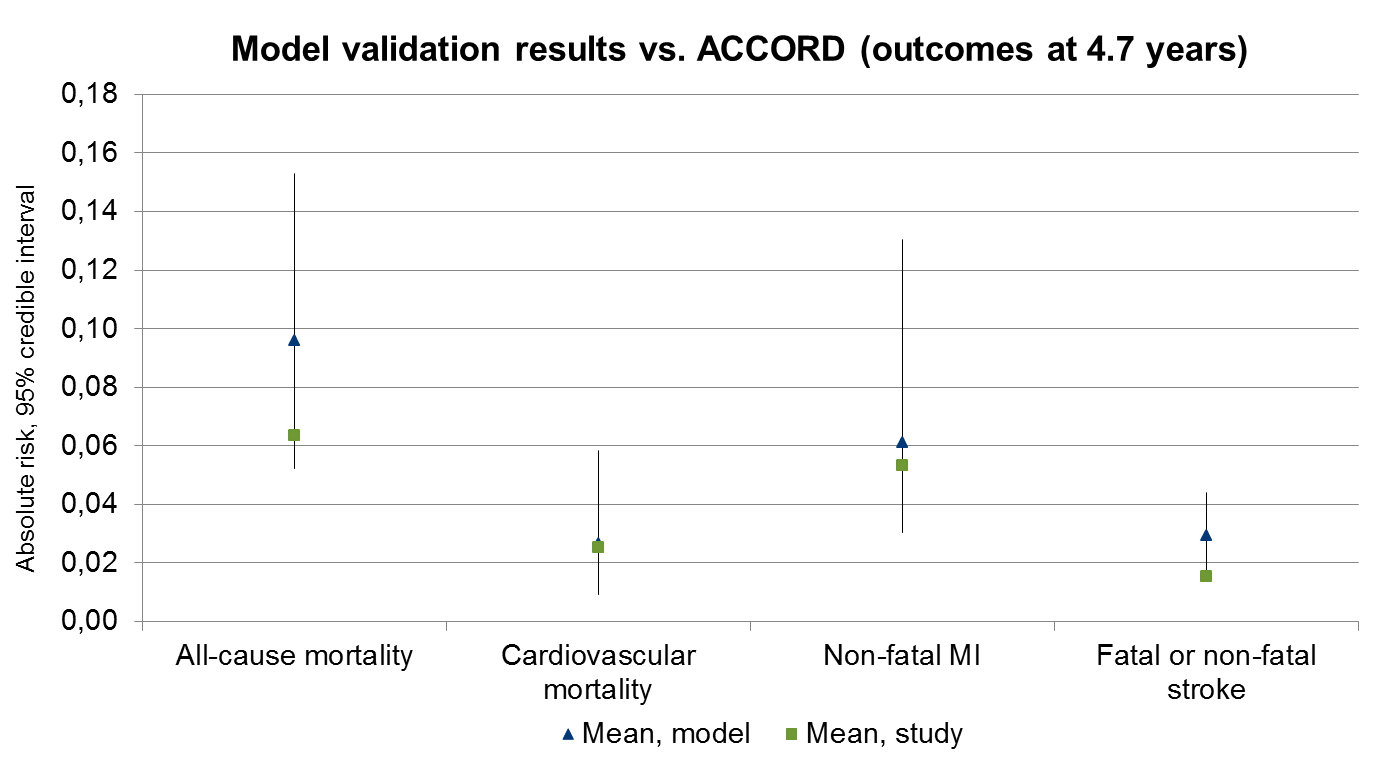


**Fig. S3** Results of model validation against ACCORD

**Validation against SOREG**

As SOREG was not used for inputs about remission and incidence of diabetes type II, it was used to validate model for these two outcomes.

Results of validation are presented in the Figures S4A and S4B.


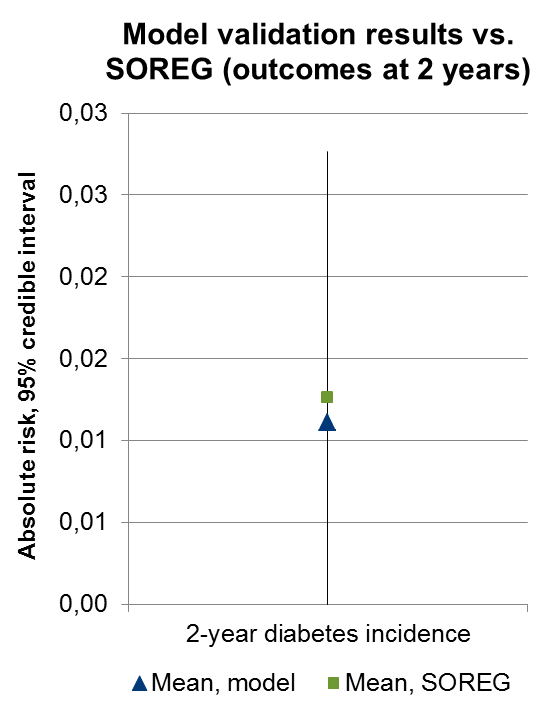


**Fig. S4A** Results of model validation against SOREG


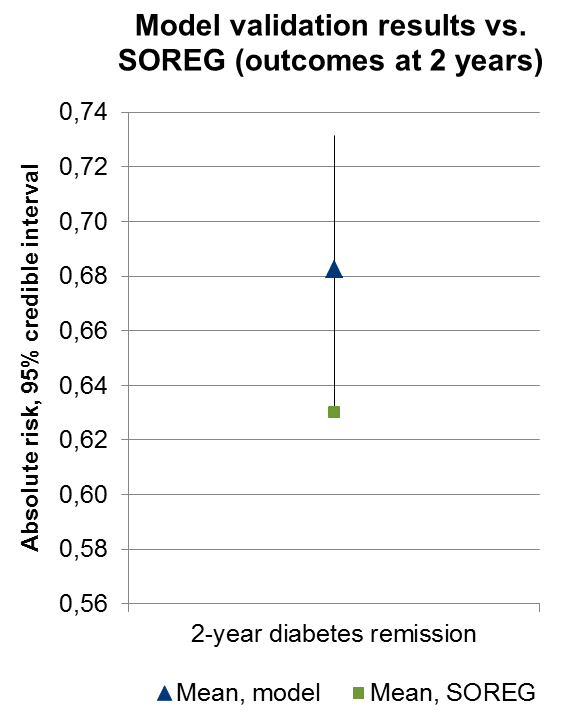


**Fig. S4B** Results of model validation against SOREG

**Section S3** Data for deterministic and probabilistic sensitivity analyses

The following scenarios were tested:

1. assuming immediate rebound of effect of bariatric surgery after 15 years after operation with BMI level reverting to level in optimal medical management arm;
2. limiting one-year impact on BMI level from very optimistic (-20 kg/m^2^) to over conservative (-4 kg/m^2^). Analysis was limited to GBP since that operation dominates the bariatric field;
3. applying different annual discount rates (costs – 5%, benefits – 5%; costs – 0%, benefits – 0%; costs – 3%, benefits – 0%);
4. removing disutility due to negative health events from analysis of health-related quality of life;
5. applying 50% reduction of health-related quality of life for month, when bariatric surgery is performed;
6. changing distribution of surgeries (percentage of GBP – 98% (current utilization), 80%, 70%, 60%; other surgeries have the same utilization);
7. changing proportion of high-volume (with more than 250 cases) centers;
8. performing analysis from societal perspective with indirect cost included;
9. using data about impact of surgery on BMI level from network meta-analysis, published in 2011 (44). In meta-analysis mean 1-year BMI reduction were -9.0 kg/m^2^ for GBP, -10.1 kg/m^2^ – for SG and -2.4 kg/m^2^ for AGB.

The following approach was used to determine distribution for variables in probabilistic sensitivity analysis:

- beta distribution was used for the probabilities;
- Gamma distributions were used for the longitudinal data, for the cost data with descriptive statistics available and for the utility data;
- lognormal distributions were used for the relative risks;
- uniform distributions were used for parameters based on expert or analyst assumptions and for parameters where descriptive statistics were not available in the original publications;
- reimbursement tariffs were not tested in probabilistic analysis.


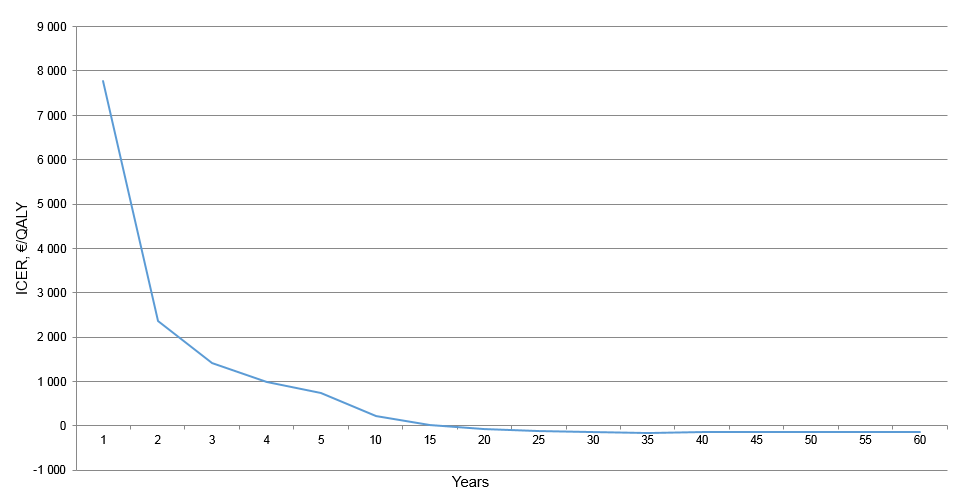


**Fig. S5** Cost-effectiveness of bariatric surgery at different time horizons

Figure shows the incremental cost-effectiveness ratio (ICER) at different time points. Swedish willingness-to-pay threshold is about €35,526/QALY. The analysis shows that, 2 years after the procedure, surgery becomes a cost-effective option. Then, the ICER curve slowly decreases which means an increase of cost-effectiveness over time. After 17 years, the curve crosses the x-axis which means that surgery becomes cost saving to the health care system.

**Table S9** Results of cost-effectiveness analysis from health care payer perspective in cohorts of male diabetic patients

|  | Cost, € | ∆ cost | LYG, years | ∆ LYG | QALY | ∆ QALY | ICER, €/QALY |
| --- | --- | --- | --- | --- | --- | --- | --- |
| Moderately obese (BMI – 33 kg/m^2^), diabetes | | | | | | | |
| OMM | 47 994 |  | 18,4 |  | 9,5 |  |  |
| Surgery | 33 014 | -14 981 | 20,1 | 1,7 | 12,9 | 3,4 | Dominates |
| Severely obese (BMI – 37 kg/m^2^), diabetes | | | | | | | |
| OMM | 48 863 |  | 18,2 |  | 8,6 |  |  |
| Surgery | 34 199 | -14 664 | 19,9 | 1,7 | 12,1 | 3,5 | Dominates |
| Morbidly obese (BMI – 42 kg/m^2^), best-case, diabetes | | | | | | | |
| OMM | 49 961 |  | 18,1 |  | 7,4 |  |  |
| Surgery | 33 928 | -16 033 | 19,9 | 1,9 | 12,3 | 4,8 | Dominates |
| Morbidly obese (BMI – 42 kg/m^2^), worst-case, diabetes | | | | | | | |
| OMM | 49 961 |  | 18,1 |  | 7,4 |  |  |
| Surgery | 34 245 | -15 716 | 19,9 | 1,8 | 12,1 | 4,7 | Dominates |
| Super obese (BMI – 52 kg/m^2^), diabetes | | | | | | | |
| OMM | 52 031 |  | 17,8 |  | 5,2 |  |  |
| Surgery | 36 618 | -15 412 | 19,6 | 1,9 | 10,7 | 5,4 | Dominates |

BMI: body mass index; ICER: incremental cost-effectiveness ratio; LYG: life years gained; OMM: optimal medical management; QALY: quality-adjusted life years.

**Table S10** Results of cost-effectiveness analysis from health care payer perspective in cohorts of male non-diabetic patients

|  | Cost, € | ∆ cost | LYG, years | ∆ LYG | QALY | ∆ QALY | ICER, €/QALY |
| --- | --- | --- | --- | --- | --- | --- | --- |
| Moderately obese (BMI – 33 kg/m2), no diabetes | | | | | | | |
| OMM | 21 654 |  | 20,3 |  | 11,9 |  |  |
| Surgery | 22 643 | 989 | 20,9 | 0,6 | 14,0 | 2,2 | 5 170 |
| Severely obese (BMI – 37 kg/m2), no diabetes | | | | | | | |
| OMM | 24 458 |  | 20,0 |  | 10,6 |  |  |
| Surgery | 24 133 | -326 | 20,8 | 0,7 | 13,1 | 2,5 | Dominates |
| Morbidly obese (BMI – 42 kg/m2), best-case, no diabetes | | | | | | | |
| OMM | 28 107 |  | 19,7 |  | 9,1 |  |  |
| Surgery | 23 797 | -4 310 | 20,8 | 1,1 | 13,3 | 4,2 | Dominates |
| Morbidly obese (BMI – 42 kg/m2), worst-case, no diabetes | | | | | | | |
| OMM | 28 107 |  | 19,7 |  | 9,1 |  |  |
| Surgery | 24 229 | -3 879 | 20,8 | 1,0 | 13,1 | 4,0 | Dominates |
| Super obese (BMI – 52 kg/m2), no diabetes | | | | | | | |
| OMM | 35 128 |  | 19,2 |  | 6,2 |  |  |
| Surgery | 27 259 | -7 869 | 20,4 | 1,3 | 11,5 | 5,3 | Dominates |

BMI: body mass index; ICER: incremental cost-effectiveness ratio; LYG: life years gained; OMM: optimal medical management; QALY: quality-adjusted life years.

**Table S11** Results of cost-effectiveness analysis from health care payer perspective in cohorts of female diabetic patients

|  | Cost, € | ∆ cost | LYG, years | ∆ LYG | QALY | ∆ QALY | ICER, €/QALY |
| --- | --- | --- | --- | --- | --- | --- | --- |
| Moderately obese (BMI – 33 kg/m2), diabetes | | | | | | | |
| OMM | 55 474 |  | 22,1 |  | 11,5 |  |  |
| Surgery | 33 230 | -22 244 | 22,9 | 0,8 | 14,8 | 3,3 | Dominates |
| Severely obese (BMI – 37 kg/m2), diabetes | | | | | | | |
| OMM | 56 540 |  | 22,0 |  | 10,4 |  |  |
| Surgery | 34 455 | -22 085 | 22,8 | 0,8 | 13,9 | 3,5 | Dominates |
| Morbidly obese (BMI – 42 kg/m2), best-case, diabetes | | | | | | | |
| OMM | 57 962 |  | 21,9 |  | 9,1 |  |  |
| Surgery | 34 153 | -23 809 | 22,8 | 1,0 | 14,1 | 5,1 | Dominates |
| Morbidly obese (BMI – 42 kg/m2), worst-case, diabetes | | | | | | | |
| OMM | 57 962 |  | 21,9 |  | 9,1 |  |  |
| Surgery | 34 426 | -23 536 | 22,8 | 1,0 | 13,9 | 4,9 | Dominates |
| Super obese (BMI – 52 kg/m2), diabetes | | | | | | | |
| OMM | 60 758 |  | 21,7 |  | 6,5 |  |  |
| Surgery | 37 213 | -23 545 | 22,7 | 1,0 | 12,4 | 5,9 | Dominates |

BMI: body mass index; ICER: incremental cost-effectiveness ratio; LYG: life years gained; OMM: optimal medical management; QALY: quality-adjusted life years.

**Table S12** Results of cost-effectiveness analysis from health care payer perspective in cohorts of female non-diabetic patients

|  | Cost, € | ∆ cost | LYG, years | ∆ LYG | QALY | ∆ QALY | ICER, €/QALY |
| --- | --- | --- | --- | --- | --- | --- | --- |
| Moderately obese (BMI – 33 kg/m2), no diabetes | | | | | | | |
| OMM | 19 600 |  | 22,7 |  | 13,3 |  |  |
| Surgery | 19 714 | 114 | 23,1 | 0,4 | 15,5 | 2,2 | 574 |
| Severely obese (BMI – 37 kg/m2), no diabetes | | | | | | | |
| OMM | 23 043 |  | 22,6 |  | 12,0 |  |  |
| Surgery | 21 239 | -1 804 | 23,1 | 0,5 | 14,6 | 2,7 | Dominates |
| Morbidly obese (BMI – 42 kg/m2), best-case, no diabetes | | | | | | | |
| OMM | 27 768 |  | 22,4 |  | 10,3 |  |  |
| Surgery | 20 875 | -6 893 | 23,1 | 0,7 | 14,8 | 4,5 | Dominates |
| Morbidly obese (BMI – 42 kg/m2), worst-case, no diabetes | | | | | | | |
| OMM | 27 768 |  | 22,4 |  | 10,3 |  |  |
| Surgery | 21 277 | -6 491 | 23,1 | 0,7 | 14,6 | 4,3 | Dominates |
| Super obese (BMI – 52 kg/m2), no diabetes | | | | | | | |
| OMM | 37 222 |  | 22,0 |  | 7,2 |  |  |
| Surgery | 24 795 | -12 427 | 22,9 | 0,9 | 13,0 | 5,8 | Dominates |

BMI: body mass index; ICER: incremental cost-effectiveness ratio; LYG: life years gained; OMM: optimal medical management; QALY: quality-adjusted life years.


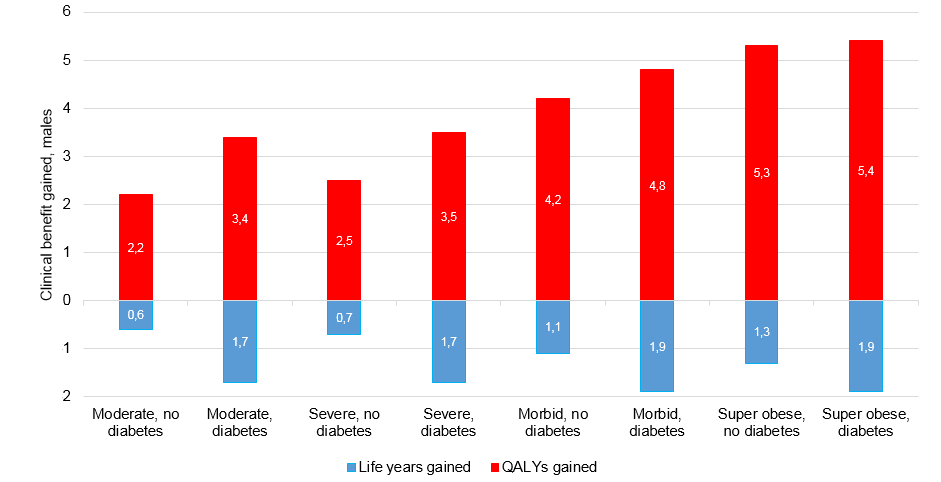


**Fig. S6A** Clinical benefits in cohorts of male patients

The figure shows the number of additional life years and quality-adjusted life years in the surgical arm in comparison with optimal medical management.


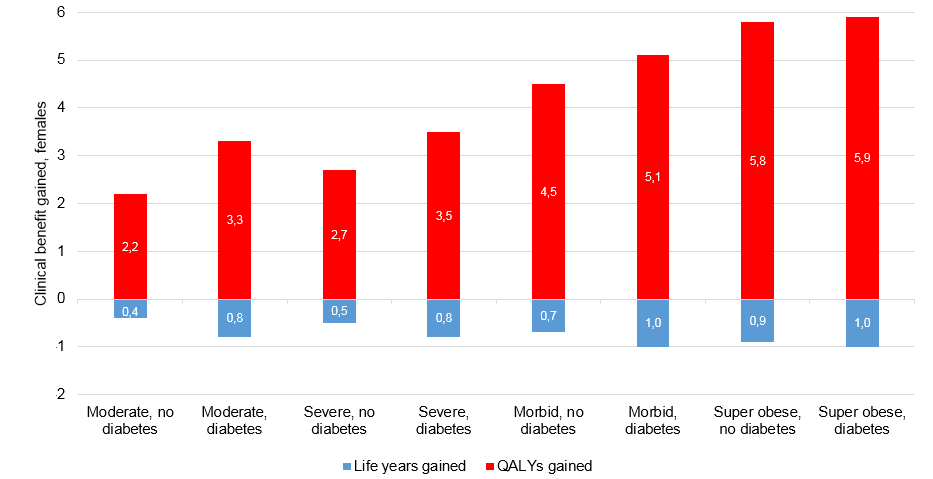


**Fig.** **S6B** Clinical benefits in cohorts of female patients

The figure shows the number of additional life years and quality-adjusted life years in the surgical arm in comparison with optimal medical management.

**Table S13** Total cost, life years and QALYs gained with no delay and 3-year delay in surgery provision

| Delay in surgery | Moderately obese | | Severely obese | | Morbidly obese | | Super obese | |
| --- | --- | --- | --- | --- | --- | --- | --- | --- |
|  | Males | Females | Males | Females | Males | Females | Males | Females |
| Total cost, € | | | | | | | | |
| Non-diabetic | | | | | | | | |
| None | 22 690 | 19 755 | 24 175 | 21 278 | 23 841 | 20 914 | 27 316 | 24 847 |
| 3-year | 22 253 | 19 307 | 23 736 | 20 811 | 23 867 | 20 908 | 27 512 | 25 017 |
| Diabetic | | | | | | | | |
| None | 33 083 | 33 300 | 34 266 | 34 525 | 33 996 | 34 224 | 36 695 | 37 291 |
| 3-year | 35 228 | 36 008 | 36 328 | 37 150 | 36 295 | 37 027 | 38 761 | 39 842 |
| Life years gained | | | | | | | | |
| Non-diabetic | | | | | | | | |
| None | 20.9 | 23.1 | 20.8 | 23.1 | 20.8 | 23.1 | 20.4 | 22.9 |
| 3-year | 20.8 | 23.1 | 20.6 | 23.0 | 20.6 | 23.0 | 20.2 | 22.8 |
| Diabetic | | | | | | | | |
| None | 20.1 | 22.9 | 19.9 | 22.8 | 19.9 | 22.8 | 19.6 | 22.7 |
| 3-year | 19.5 | 22.8 | 19.3 | 22.8 | 19.3 | 22.8 | 19.0 | 22.6 |
| Quality-adjusted life years gained | | | | | | | | |
| Non-diabetic | | | | | | | | |
| None | 14.0 | 15.5 | 13.1 | 14.6 | 13.3 | 14.8 | 11.5 | 13.0 |
| 3-year | 13.7 | 15.3 | 12.8 | 14.3 | 12.7 | 14.3 | 10.7 | 12.3 |
| Diabetic | | | | | | | | |
| None | 12.9 | 14.8 | 12.1 | 13.9 | 12.3 | 14.1 | 10.7 | 12.4 |
| 3-year | 12.2 | 14.4 | 11.4 | 13.5 | 11.3 | 13.5 | 9.5 | 11.6 |

**Section S4** Results of Scenario Analysis

Results of the scenario analyses are presented below:

1. *assuming the improbable immediate rebound of the effect of bariatric surgery 15 years after the operation with a BMI level reverting to the level of the optimal medical management arm*;

The rebound effect (assuming that the BMI level will be reverted to the BMI level in optimal medical management arm after 15 years) provides a moderate impact on the level of cost savings (from €3082 with no rebound to €1379 with a rebound effect) and clinical benefits (life years gained from 0.9 to 0.7 and QALYs from 3.5 to 2.4 with no rebound and rebound effect, respectively).

1. *varying the one-year impact on the BMI level*;

In the present analysis, the impact of potentially very high (20 kg/m^2^ reduction at one year) or unrealistically low (4 kg/m^2^ reduction at one year) weight-loss effect on the cost-effectiveness of surgery was tested. The analysis showed that with unrealistically low effect (reduction of 4 kg/m^2^ one year post-GBP), surgery produces lifetime benefits (0.2 life years and QALYs) and remains cost-effective in Swedish settings (ICER of €21,097/QALY). Overall, realistic change of the effect of surgery does not affect its economic/clinical position.

1. *applying different annual discount rates for costs and benefits*;

In the base-case analysis, a 3% discount rate for cost and benefits was applied. It means that with each subsequent year included in the model, the value of cost and QALYs incurred would be depreciated by 3%. With changes of discount rate, results remained stable. Cost savings and clinical benefits were maximized with a zero discount rate for both cost and effect. Details are provided in *Table S14*.

1. *removing from the analysis of health-related quality of life disutility of adverse health events*;

In the base-case analysis, disutility of adverse health events (i.e., stroke) is included. The removal of disutility had a limited impact on the level of QALY gained: 3.45 *versus* 3.52 with disutility excluded and included, respectively.

1. *applying a 50% reduction of health-related quality of life for the month of surgery;*

Acknowledging the impact of primary surgery on health-related quality of life led only to a 0.02 QALY loss of the overall QALY gained in the surgery arm and, therefore, it does not influence the results significantly.

1. *changing the distribution of surgeries;*

The reduction of share of GBP (from current 98%) with corresponding proportional increase of SG and GP resulted in decreases of cost savings (98% - minus €3082; 80% - minus €2520; 70% - minus €2223; 60% - minus €1925) and clinical benefits (98% - 3.5 QALYs; 80% - 3.2 QALYs; 70% - 3.1 QALYs; 60% - 2.9 QALYs).

1. *changing the proportion of high-volume centres*;

Changing the proportion of high-volume centres had a limited impact on the overall cost of surgery as the cost of primary surgery only contributes 22% to the overall lifetime cost in the surgical arm. Details are provided in *Fig. S7-8.*

1. *performing the analysis with indirect costs included*;

When indirect costs (absenteeism from work due to sickness and early retirement) were included in the analysis, the total cost in the surgical arm was €38,822 *versus* €54,789 in the optimal medical management arm. Overall, the cost savings increased from €7798 in the analysis from the health care payer perspective to €15,967 in the analysis from the societal perspective.

1. *using data from a network meta-analysis on the impact of surgery on the BMI level;*

In the analysis using conservative effectiveness inputs^(44)^ and similar patient characteristics to the base-case scenario, surgery was still cost saving. Over a lifetime, it led to savings of €3423, a gain of 0.5 life years and 2.0 QALYs per patient.

In general, extensive sensitivity and scenario analyses showed that the uncertainty around the model inputs and structure did not affect the main results significantly.

**Table S14** Sensitivity analysis for annual discounting rate

|  | Cost, € | ∆ cost | LYG, years | ∆ LYG | QALY | ∆ QALY | ICER, €/QALY |
| --- | --- | --- | --- | --- | --- | --- | --- |
| Base-case analysis (3% cost and benefits) | | | | | | | |
| OMM | 49 501 |  | 19,7 |  | 8,8 |  |  |
| Surgery | 39 617 | -9 884 | 20,6 | 0,9 | 12,3 | 3,5 | Dominates |
| 0% cost and benefits | | | | | | | |
| OMM | 87 153 |  | 31,0 |  | 13,7 |  |  |
| Surgery | 66 959 | -20 194 | 33,3 | 2,3 | 19,8 | 6,1 | Dominates |
| 5% cost and benefits | | | | | | | |
| OMM | 35 711 |  | 15,5 |  | 7,0 |  |  |
| Surgery | 29 921 | -5 790 | 16,0 | 0,5 | 9,6 | 2,6 | Dominates |
| 3% cost and 0% benefits | | | | | | | |
| OMM | 49 501 |  | 31,0 |  | 13,7 |  |  |
| Surgery | 39 617 | -9 884 | 33,3 | 2,3 | 19,8 | 6,1 | Dominates |

BMI: body mass index; ICER: incremental cost-effectiveness ratio; LYG: life years gained; OMM: optimal medical management; QALY: quality-adjusted life years.


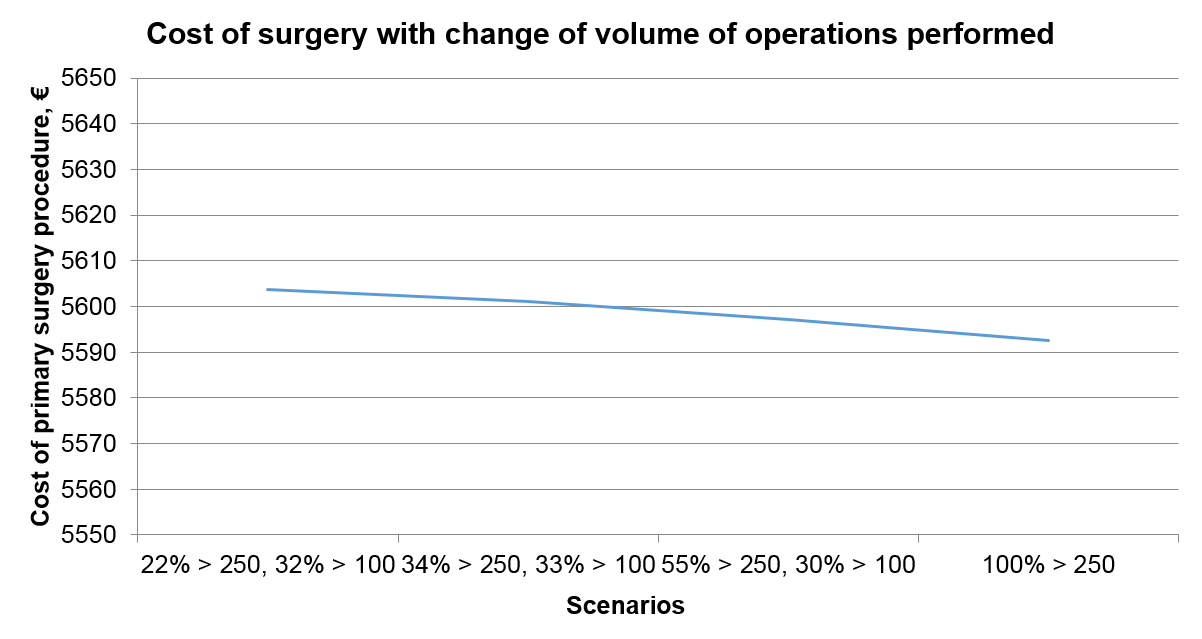


**Fig. S7** Impact of proportion of high-volume hospitals on cost of bariatric surgery


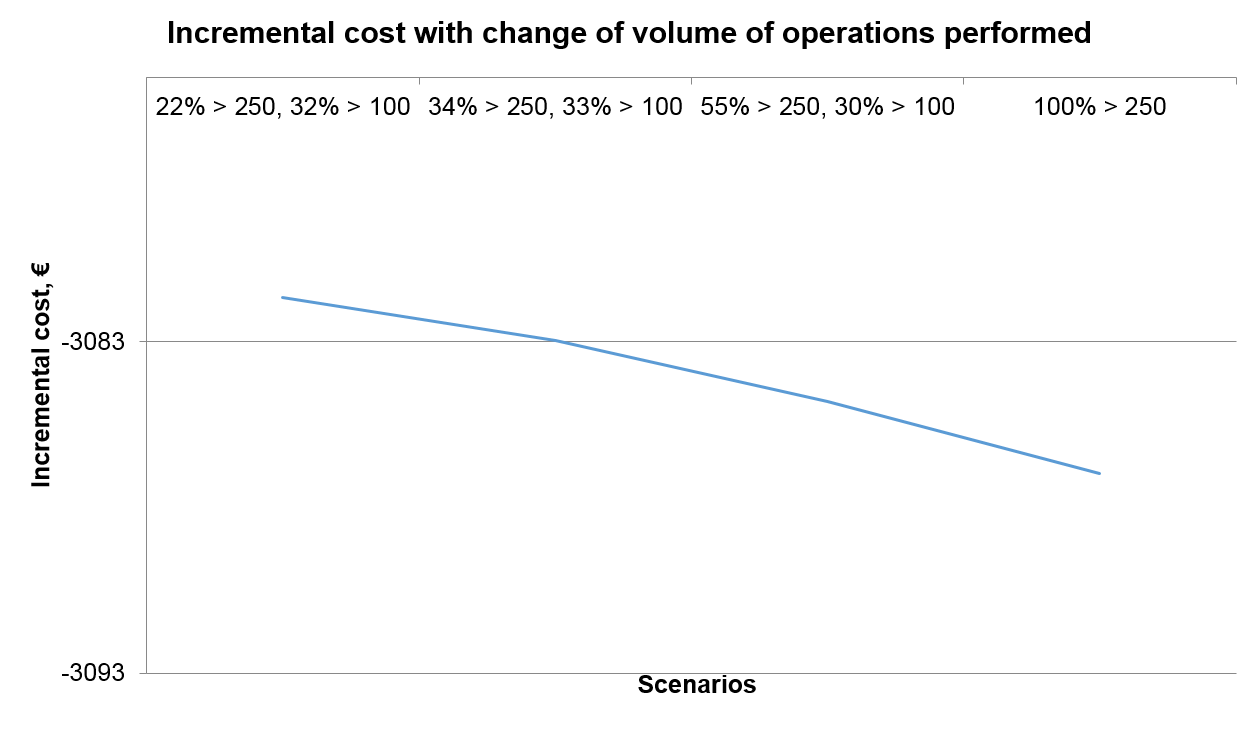


**Fig. S8** Impact of proportion of high-volume hospitals on incremental cost of bariatric surgery
